# Pleiotropic Bioactivity of Caterpillar Fungus, Orange Cordyceps, and Cordycepin: Insight from Integrated Network Pharmacology and Food and Drug Regulatory Framework

**DOI:** 10.3390/ph19030519

**Published:** 2026-03-23

**Authors:** Alexander Panossian

**Affiliations:** Research & Development, Phytomed AB, 59344 Västervick, Sweden; ap@phytomed.se

**Keywords:** *Ophiocordyceps sinensis*, *Cordyceps militaris*, cordycepin, adaptogen, resilience, fungi, botanicals, network pharmacology

## Abstract

**Background/Objectives:** The medical mushroom *Ophiocordyceps sinensis* (Caterpillar Fungus), known for its ability to enhance “vitality,” is one of the most popular medicines in Asian traditional medical systems. According to the Chinese *Pharmacopeia*, *O. sinensis* is standardized for its adenosine content, the precursor of ATP, which mediates numerous physiological and pathological processes in many diseases. The related fungus of order Hypocreales, *Cordyceps militaris*, and its major bioactive constituents, 3′-deoxyadenosine (cordycepin), also exhibit pleiotropic biological activities. This review aims to provide a rationale for the adaptogenic and resilience-supporting effects of these medicinal fungi and to align food and drug regulation in Western countries. **Methods:** In this narrative review, we integrated results from chemical, pharmacokinetic, network pharmacology, preclinical, and clinical studies of *O. sinensis*, *C. militaris*, and cordycepin using network pharmacology and bioinformatics tools. **Results:** Across studies, recurrent mechanistic hubs included PI3K–Akt, AMPK–mTOR, MAPK, NF-κB, apoptosis, and adaptive stress-response signaling pathways, linking immune regulation and metabolic homeostasis. Experimental studies confirmed modulation of cytokine production, kinase signaling, and mitochondrial regulators. Clinical meta-analyses demonstrate consistent adjunctive benefits in renal and pulmonary disorders, although heterogeneity in preparation and methodological limitations remains significant. The review reveals controversy regarding the bioavailability of cordycepin in vivo and its concentration in vitro studies, raising the hypothesis that cordycepin may act as a driver, triggering the organism’s adaptive stress response in stress-induced and aging-related diseases. Pharmacokinetic data indicate that systemic cordycepin concentrations after oral administration remain in the nanomolar range, suggesting that some predicted molecular interactions may occur indirectly or through systems-level mechanisms. The review, for the first time, suggests establishing a regulatory category for resilience-supporting physiological modulators to align food and drug regulation in the EU with contemporary systems biology, thereby complementing the work of EFSA, EMA, FDA, and Asian authorities. **Conclusions:** *O. sinensis*, *C. militaris*, and 3-deoxyadenosine share a common adaptogenic mechanism for maintaining homeostasis of cellular and integrated biological system functions. The systems-level network analysis and reductionistic molecular ligand preceptor pharmacology provide complementary approaches for understanding the multi-target bioactivity of these fungi. This review clarifies conceptual and regulatory barriers to recognizing resilience-supporting interventions and informs future regulatory innovation.

## 1. Introduction

*Ophiocordyceps sinensis* (Berk.) G.H. Sung, J.M. Sung, Hywel-Jones and Spatafora, 2007 (Caterpillar Fungus) [1] and *Cordyceps militaris* (L.) Fr., 1818 (Orange Cordyceps) [2] are botanicals [3] belonging to the fungi kingdom [4,5,6], traditionally valued across East Asia [7,8,9,10,11,12] for their tonic, energy-enhancing, aphrodisiac, and respiratory ailments, lung invigoration, and kidney-nourishing actions [13,14,15,16,17,18,19], Appendix A Table A1 and Table A2. Their therapeutic applications span inflammatory, respiratory disorders, fatigue, metabolic diseases (atherosclerosis, hyperlipidemia, glucose metabolism), kidney diseases, fertility, sexual-restorative functions, which are associated with immune, nervous, endocrine, cardiovascular, respiratory, renal, hepatic systems [14,20,21,22,23,24,25]. These attributes align with the modern concept of adaptogens as a therapeutic category of herbal medicines and nutritional products, characterized by increased human adaptability, survival, and resilience in response to stress by triggering intracellular and extracellular adaptive signaling pathways within cellular and organismal defense systems, including the neuroendocrine-immune complex [26]. In 1968, the term adaptogen (phytoadaptogen) was applied to medicinal plants, herbal medicines, and phytomedicines, reflecting their origin in the plant kingdom [27]. Phytoadaptogens are plant secondary metabolites, particularly tetracyclic terpenoids, and phenethyl- or phenylpropanoid derivatives of the defense system, chemically similar to steroidal and phenolic hormones of the human stress system [28].

Several studies claim that *O. sinensis* is an adaptogen [29,30,31,32] that triggers various adaptive stress–response signaling pathways [33,34,35,36,37,38]. However, the rationale for polyvalent action and the molecular mechanisms of *O. sinensis* have not been sufficiently studied or understood. Furthermore, clinical efficacy was not unambiguously demonstrated, and potential new indications for *O. sinensis* should be evaluated.

Unlike phytoadaptogens derived from the green plant kingdom Viridiplantae, *O. sinensis* [1,2,5,39,40], commonly known as Caterpillar Fungus or Winter Worm-Summer Grass, is an entomopathogenic fungus of the phylum Ascomycota, with a quite different biological nature compared to typical edible mushrooms (Basidiomycota). Initially described as *Sphaeria sinensis* by Berkeley in 1843, it was later named *Cordyceps sinensis* by Saccardo in 1878, and in 2007 it was renamed *Ophiocordyceps sinensis*, a member of the genus *Ophiocordyceps* (family Ophiocordycipitaceae, order Hypocreales), based on phylogenetic studies [3,4,10,25].

Caterpillar Fungus symbiotically parasitizes the larvae of ghost moths (*Fam. Hepialidae*, *Hepialus* spp./*Thitarodes* spp.) to survive, producing a characteristic club-shaped stroma that emerges from the mummified caterpillar [13,14,16,21,29,30,41,42]. As the larva’s self-defense mechanism weakens, the fungal cells spread throughout the body, forming the sexual stroma, which grows upward above the soil surface while remaining connected to the dead larva/host below, creating the unique insect/fungus combination [43,44,45,46].

**Figure 1:**
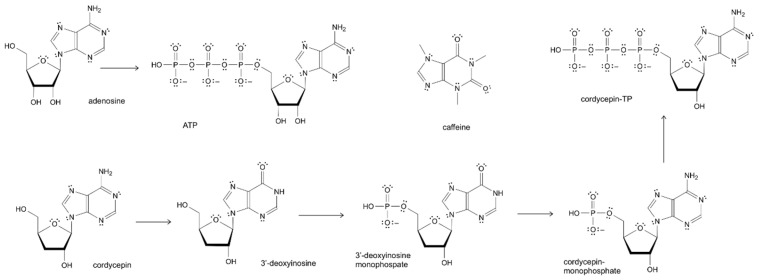
Chemical structure of purine alkaloids adenosine, cordycepin, 3′-deoxyinosine, and phosphorylated nucleoside ATP, cordycepin-TP, 3′-deoxyinosine-monophosphate. (primary metabolites of *O. sinensis* and *C. militaris*) and caffeine (a comparator-major active constituent of coffee and green tea).

This medicinal fungus is endemic to high-altitude regions of the Tibetan Plateau and the Himalayas [47,48] and has a long history in traditional medical systems of China, Nepal, India, Bhutan, Republic of Korea, Vietnam, Thailand, and Japan as a tonic to replenish bodily health [19,39]. Caterpillar Fungus is officially recognized as a medicinal fungus in the pharmacopeias of several countries, especially in East Asia, where it is highly valued for energy, immune, and respiratory support [49]. In *Pharmacopeia* of the People’s Republic of China, Chinese Caterpillar Fungus, Cordyceps (Dongchongxiacao) is classified as a *Chinese Materia Medica* crude drug, indicated for deficiency of kidney essence, impotence and seminal emission, limp aching in the lower back and knees, chronic cough and dyspnea of deficiency type, cough caused by consumptive diseases, and hemoptysis in daily dosage 3–9 g corresponding to ~1 mg of adenosine [50], Figure 1.

Related species, *C. militaris* [2], also known as the Scarlet Caterpillar Club and in Chinese as Northern Cordyceps, is cultivated on silkworm pupae and used for medicinal purposes, as a health supplement, and as a pharmaceutical drug on a large scale, especially in China [25,51]. Numerous studies on the chemical constituents and pharmacological activities of *C. militaris* [20,22,52,53,54] and its bioactive marker, cordycepin [53,54], reveal multiple effects on the neuroendocrine-immune complex [36,37,42,55,56,57,58,59,60,61,62], which is typical of adaptogens [22]. In the course of the search for new fungal antibiotics in 1950, Cunningham et al. found that the mold *C. militaris* secretes a substance called cordycepin, which inhibits the growth of several bacteria [63], increases the survival time of mice with Ehrlich ascites tumors, and inhibits the growth of human tumor cells in culture. In 1980, the chemical structure of cordycepin was elucidated as the nucleoside 3′-deoxyadenosine [64], a copy of adenosine with one oxygen atom removed from its structure (Figure 1).

**Figure A1:**
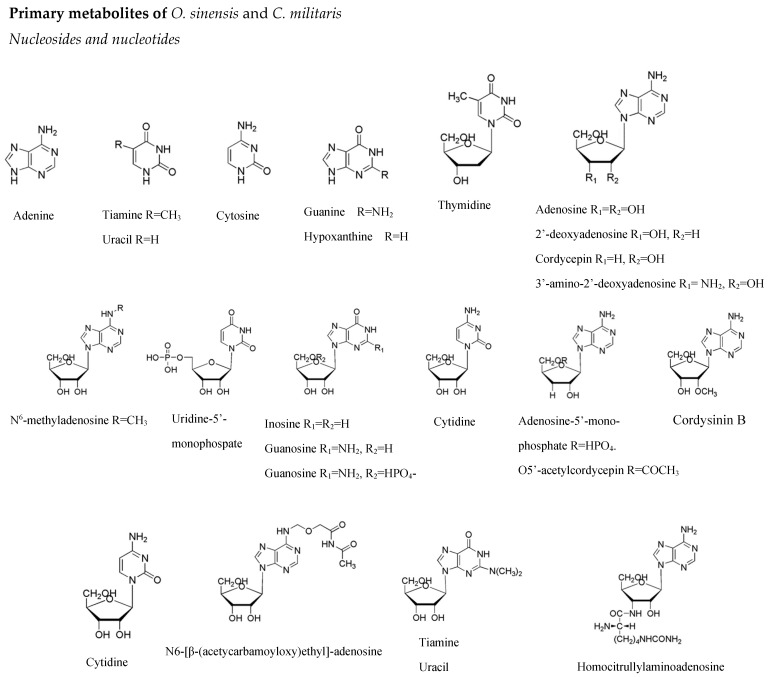
Primary metabolites: nucleosides and nucleotides.

Both fungi contain diverse bioactive metabolites, including nucleosides, sterols, peptides, polysaccharides, and secondary metabolites (Appendix A Figure A1 and Table A3, Table A4, Table A5 and Table A6).

The pharmacological breadth of both species suggests multi-target effects rather than classical single-receptor drug actions (Appendix A Table A4 and Table A5). Network pharmacology has become a significant framework for elucidating the complex, multi-target actions of such herbal and fungal preparations, providing a systematic approach to deciphering these interactions by integrating predicted targets, signaling pathways, molecular docking, and multi-omics data. However, most reports remain scattered, disease-specific, or lacking integration with wet-lab validation. A unified synthesis is needed to understand the core pharmacological principles of *O. sinensis* and *C. militaris*.

Despite numerous studies, no comprehensive synthesis has unified the mechanistic insights across species, compounds, and disease contexts. This narrative study integrates 37 network pharmacology investigations and 10 experimental validation studies to define the adaptogenic potential and systems-level pharmacology of these fungi and their active constituent, 3-deoxyadenosine (cordycepin).

Network pharmacology provides a valuable framework for understanding these multi-component, multi-target interactions. Nevertheless, existing studies are often disease-specific and fragmented, lacking an integrated mechanistic synthesis. This work consolidates findings from 37 network pharmacology studies, including 22 experimentally validated investigations, to establish a unified systems-level model for the adaptogenic activity of Caterpillar Fungus, Orange Cordyceps, and 3-deoxy-adenosine.

The adaptogenic concept represents a systems-level interpretation of complex physiological responses characteristic of holistic Traditional Chinese Medicine (TCM) and Ayurvedic concepts, whereas European Food Safety Authority (EFSA) regulatory frameworks lack concepts for resilience and adaptive capacity and operate at the level of single, well-defined functional outcomes, which raises regulatory issues for food and drugs in Western countries.

The implementation of European standards by EMA drug regulatory authorities for traditional herbal medicines and other botanicals developed under regulatory conditions in Asian countries creates problems for their regulation in Europe. This is mainly due to the core conceptual differences between reductionistic, mechanistic, and Systems-based, integrative, and holistic approaches to medicine in Western and Oriental countries, as well as to insufficient clinical evidence and limited well-established use. Another limitation in EU countries is that the EFSA does not accept the definitions of stress, fatigue, and adaptogens as dietary supplements or food additives, despite their worldwide scientific recognition. The reason is that EFSA considers these products for use only in healthy subjects, whereas medicines are used to treat or prevent defined diseases. However, the same products are used in China, India, Japan, and other Asian countries to restore balance and resilience. In this review, we sought to harmonize these complementary paradigms to address the gap and reach consensus.

Overall, this literature review primarily aims to justify a rationale for the pleiotropic adaptogenic potential of two traditionally used botanicals from the fungal species, Caterpillar Fungus and Orange Cordyceps, and its active constituent, 3-deoxyadenosine (cordycepin), using a network pharmacology approach The secondary aim was to establish a regulatory category for resilience-supporting physiological modulators that could align food and drug regulation in the EU with contemporary systems biology, and regulatory acceptance of adaptogenic botanicals across the European Union (EU), United States (US), and selected Asian jurisdictions.

## 2. Results

### 2.1. Bioactive Compounds of O. sinensis and C. militaris Fungi and Their Pharmacological Activity

Overall, 90 primary and secondary metabolites in *O. sinensis* and 26 constituents in *C. militaris* were identified in an extensive review, covering its traditional uses, chemistry, and pharmacology [19], as shown in Appendix A Table A3 and Table A4 and Figure A1.

Studies of chemical compositions of *O. sinensis* [21,29,65] and *C. militaris* [23,29,66,67] show the presence of host–parasite symbiotic interactions, four types of primary metabolites: (i)—amino acids and peptides, (ii)—nucleotides/nucleosides, (iii)—lipids, including sterols and fatty acids, and (iv)—carbohydrates, including polysaccharides. All of them are essential for the parasite’s growth and development, as well as for the host’s adaptive stress response (Appendix A Table A4, Table A5 and Table A6). Some secondary metabolites that function in defense responses include cytotoxic alkaloids and antioxidant phenolic compounds. These compounds were isolated and identified in *O. sinensis* growing under wild conditions, but not in cultivated *O. sinensis* and *C. militaris* (Appendix A Figure A1).

Many primary metabolites of mushrooms, including nucleosides, sterols, and polysaccharides, have been specified as physiologically active markers of *O. synensis* in both in vitro and in vivo studies [68]. Meanwhile, several novel compounds characteristic of *O. sinensis* have been identified, including epipolythiodioxopiperazines, gliocladicillins A, B, and 11,11′-dideoxyverticillin, which are capable of inhibiting the growth of tumor cells [16,69,70]. Exclusive to *O. sinensis*, five anti-inflammatory alkaloids, named cordysinin (A–E), have also been reported for the first time [16,19,71].

At present, cordycepin, adenosine, total nucleosides, ergosterol, and polysaccharides are commonly used quality control markers for *O. sinensis* and *C. militaris* products (Table 1 and Table A5) [18,71,72,73,74,75,76,77,78,79,80,81,82,83,84,85,86,87,88,89,90,91,92,93,94,95,96,97]. However, they are not regarded as efficacy markers for therapeutic or nutritional purposes for several reasons, including poor oral bioavailability, instability, nonspecific (cordycepin, adenosine) or inconsistent actions (polysaccharides, ergosterol), and a lack of direct correlation between marker levels and clinical effects in humans [84,85].

*O. sinensis* exhibits broad biological and pharmacological actions in hepatic, renal, cardiovascular, and immunological systems, and has anticancer activity as well [15,97]. Over 30 different bioactivities have been reported for *O. sinensis*, including anti-inflammatory, immunomodulatory, antioxidant, antibacterial, hepatoprotective, antifatigue, antiaging, steroid hormones production, antidepressant, sedative, and the ability to promote endurance capacity, improve learning-memory in vitro, in vivo, or ex vivo studies, as shown in Appendix A Table A4 [97].

In the course of the search for new fungal antibiotics in 1950, Cunningham et al. found that the mold *C. militaris* secretes a substance called cordycepin, which inhibits the growth of several bacteria [63], increases the survival time of mice with Ehrlich ascites tumors, and inhibits the growth of human tumor cells in culture. In 1980, the chemical structure of cordycepin was elucidated as the nucleoside 3′-deoxyadenosine [64] (Figure 1). One of its modes of action is by inhibition of RNA synthesis. It is incorporated as 3′-deoxyadenosine triphosphate at the 3′ end of the RNA molecule, thereby preventing further elongation [98]. Phosphorylated adenosine, adenosinetriphosphate (ATP), is known as an “energy currency” in the metabolism of the organism. A cellular energy sensor, AMPK (AMP-activated protein kinase (PRKAA1/2/PRKAG1) is one of the key mediators of adaptive stress–response signaling pathways. Phosphorylated cordycepin (cordycepin triphosphate, COR-tp) competes with ATP, resulting in its incorporation into enzymatic processes where it acts as a substitute for ATP [99,100]. This molecular mimicry may underlie the diverse biological activities of cordycepin, leading to abnormal purine metabolism and inhibiting or aberrantly activating ATP-targeted protein kinases [35,62]. Consequently, it is not surprising that cordycepin may have beneficial health effects in stress-induced metabolic diseases and aging disorders, showing anticancer, antiviral, antioxidant, anti-aging, and anti-inflammatory activities [33,34,35,36,37,38,42,55,61,101,102,103,104].

Overall, the pharmacological activity of *O. sinensis* and *C. militaris* product preparations is unlikely to be attributable to a single active constituent, as compounds such as cordycepin and adenosine suffer from poor oral bioavailability and limited pharmacokinetic stability. Instead, it is reasonable to hypothesize that the overall therapeutic profile arises from the synergistic action of multiple metabolites—including nucleosides, polysaccharides, sterols, and peptides—that interact with diverse molecular targets. At present, the validity of this hypothesis rests primarily on the outcomes of randomized controlled clinical trials, rather than on any single chemical marker. In this context, network pharmacology approaches combined with gene expression analyses may be suitable tools for elucidating the complex, multi-target interactions underlying the putative synergistic effects of *O. sinensis* and *C. militaris* ingredients.

### 2.2. Nucleosides Adenosine and Cordycepin as Mediators of Adaptive Stress Response Andanti-Fatigue Activity of O. sinensis, and C. militaris

The choice of adenosine as an active marker for the standardization of Cordyceps (Dongchongxiacao), Chinese Caterpillar Fungus [50] and Patented Traditional Chinese Medicines (TCM) Bailing capsule [105], containing fermented *O. sinensis* powder standardized for 0.4 mg of adenosine, is presumably due to: (i) its high content in *O. sinensis* and (ii) its physiological functions.

Adenosine functions as an energy metabolite (part of the ATP/AMP balance) and as a signaling molecule via cell-surface receptors and intracellular actions in the nervous, cardiovascular, immune, respiratory, and renal systems [106,107,108,109,110,111]. Adenosine is a byproduct of the “energy fuel”, adenosine triphosphate (ATP) breakdown during stress (hypoxia, ischemia, exercise), matching oxygen supply with demand as a local, short-lived homeostatic regulator, and coordinating energy balance, vascular tone, neuronal excitability, and immune activity [112,113,114,115,116,117,118]. During low-energy states, adenosine acts as a local signal to increase energy supply. Adenosine indirectly regulates AMP-activated protein kinase (AMPK) activity through AMP/adenosine balance. High AMP/ATP ratios activate AMP-activated protein kinase (AMPK), promoting energy-conserving processes. Adenosine can also directly activate receptors, and its balance with AMP is maintained by enzymes such as adenosine kinase (ADK), as shown in Figure 1. Its primary molecular targets are the four adenosine GPCRs (A1, A2A, A2B, A3), as well as metabolic enzymes and transporters that control its levels [117,119,120,121,122].

Adenosine is the endogenous agonist of GPCR adenosine receptors (A1, A2A, A2B, A3, high affinity; EC50 in the low-nanomolar range), producing inhibitory neuromodulation that promotes sleep, decreases neuronal firing, and increases the subjective sensation of fatigue [101,123], Table 2. In contrast, caffeine acts as a competitive antagonist at A1 and A2A receptors (non-selective; micromolar affinities, Ki ~2–50 µM), thereby blocking adenosine’s inhibitory signaling and producing stimulatory effects on arousal, mood, reduced fatigue, and physical performance [124,125,126], Table 2. Cordycepin (3′-deoxyadenosine), despite structural similarity to adenosine, can act as an agonist in vitro (at A3 in many cell studies and A1/A2A in some reports), and has minimal functional interaction with adenosine receptors in vivo because it is rapidly deaminated to 3′-deoxyinosine, exhibits very low plasma nano-molar levels after oral ingestion [127,128], and demonstrates poor blood–brain barrier penetration, meanwhile in cell/animal models cordycepin’s effects (anti-inflammatory, antiproliferative) are often attributed to A3 agonism or other intracellular actions (AMPK activation, PI3K/mTOR inhibition) [101,129,130]. Consequently, cordycepin does not meaningfully modulate adenosine receptor activity and cannot reproduce either the inhibitory effects of adenosine or the stimulatory effects of caffeine. The net result is that adenosine is fatigue-promoting, caffeine is fatigue-reducing, and cordycepin is functionally neutral in the context of central fatigue regulation (Table 2).

Cordycepin cannot meaningfully act as a CNS adenosine receptor ligand, yet *O. sinensis* and *C. militaris* extracts and purified cordycepin repeatedly show anti-fatigue effects in animals and humans. This appears contradictory only if one assumes that fatigue modulation must occur via central adenosinergic signaling. Apparently, the anti-fatigue effects of *O. sinensis* and *C. militaris* are mediated by non-adenosinergic, non-CNS mechanisms, though somewhat peripheral.

Cordycepin has poor intestinal permeability and low bioavailability due to rapid hepatic degradation by the enzyme adenosine deaminase, which converts it to the inactive 3′-deoxyinosine. However, 3′-deoxyinosine has significant bioavailability following oral administration of cordycepin, when absorbed into the systemic circulation, and can be phosphorylated to the active cordycepin 5′-triphosphate, an analog of ATP [131], Figure 1, and therefore could be responsible for the therapeutic effects of cordycepin when administered orally. These findings offer important insights into the mechanisms underlying the therapeutic effects of cordycepin. Moreover, this metabolic pathway could play an important role in the activity of adenosine and other adenosine analog drugs. The metabolized products (i.e., the inactive metabolite of cordycepin, 3′-deoxyinosine) are available in the systemic circulation instead. They can return to the phosphorylation pathway of their parent form, as shown in this study [131].

The bioavailability of cordycepin is very low; a 10 mg/kg intravenous dose in rats yields a peak concentration of 2.1 ± 0.9 μg/mL (8366 nM). However, when cordycepin is administered orally to rats at a 10-fold higher dose of 100 mg/kg, it results in a relatively low peak plasma concentration, averaging approximately 0.004 ± 0.001 μg/mL (16 nM) [127]. The Cmax values of cordycepin in the rat blood and brain after exposure (10 mg/kg, i.p.) were 7.8 ng/mL (31 nM) and 5.4 ng/mL (21 nM), respectively [128]. Pharmacokinetic and brain research studies [127,128,131,132] reveal that the concentration of cordicepin in blood, kidney  >  liver  >  heart  >  lung  >  spleen  >  brain is dramatically lower than observed in vitro studies [127]. After systemic dosing with 10 mg cordycepin, its concentration in rat brain tissue is on the order of ~10–50 nM [127,128], while most published microglia/astrocyte studies use 10–50 μM cordycepin, which is 1000-fold higher than the presumed brain levels from a 9 g/day *O. sinensis* crude dose. These μM levels are clearly supra-physiological compared with the nM brain levels observed in PK studies; they are more like “pharmacological screening” than strict dose translation.

Obviously, the active concentrations of cordicepin, *O. sinensis*, and *C. militaris* preparations used in vitro studies are 1000-fold higher than those used in animal studies and do not match the effective therapeutic human daily dose of 9 g of *O. sinensis* and *C. militaris* crude drugs [50], which are traditionally used.

### 2.3. Efficacy and Safety of Ophiocordyceps sinensis and Cordyceps militaris in Human Subjects

#### 2.3.1. Traditional Use

Oriental medical systems, TCM and Ayurveda, are based on a holistic approach, multi-target and polyvalent actions, but rely on archaic theories. In contrast, Western conventional medicine is based on a reductional approach, selective targeting, and specific action in the treatment of diseases, relying on another archaic theory of galenic preparations that ignores interactions and overlaps among regulatory systems and the effects of other constituents in multi-component plant extracts. In fact, both approaches are complementary, particularly in the treatment of diseases of complex pathology associated with the stress system interacting with other regulatory systems. The concomitant administration of several Western conventional medicines that selectively act on various receptors provides more effective treatment in hypertension, post-stroke rehabilitation, viral infections, etc. The “ready for use” of complex botanicals and botanical hybrid products used in the Eastern world acts similarly. The challenge is to understand the mechanisms of action and scientifically validate their efficacy, quality, and safety.

According to traditional Chinese medicine (TCM) theory, the organ functions concepts differ from Western physiology and pathology, suggesting that Caterpillar Fungus goes to the “Lung” and the “Kidney” meridians, supporting “lung protection,” “kidney improvement,” and so-called “YinYang double invigoration” [133]. Since the kidneys are considered “the root of life” in TCM, they store “Jing,” a substance described as an undifferentiated, prime organic material that is “the source of reproduction, development, and maturation” [133]. Conception is made possible by the power of Jing; growth to maturity is the blossoming of Jing; and the decline into old age reflects the weakening of Jing. Over time, the Jing decreases in both vitality and quantity. Consequently, according to TCM, reproductive problems such as sterility or impotence and developmental disorders like retarded growth or lack of sexual maturation are seen as a dysfunction of the kidney’s storing of Jing [133]. Moreover, according to TCM theory, the kidneys enable the fundamental “energy of life, so-called the Natural Air”, Qi/chi, to penetrate deeply, completing the inhalation process by what is called “grasping the Qi.” The kidneys are thus the root of Qi, while the lungs are the “foundation of Qi.” Proper breathing thus depends on the kidneys, and kidney disharmonies may result in respiratory problems, especially chronic asthma. In a “disharmony of the Lungs,” a deficient Qi can result in any area of the body, and the power of resistance of the “protective Qi will be poor” [13,133]. Both TCM and Ayurveda have a notion of “life vital energy” and activating the body and mind: the qi in TCM and the prana in Ayurveda. Herbalists refer to adaptogens as restoratives, qi-tonics, rasayanas, or rejuvenating herbs [134].

According to TCM, Caterpillar Fungus goes to the “Lung” and the “Kidney” meridians, supporting “lung protection,” “kidney improvement,” and so-called “YinYang double invigoration” [133] and therefor was used to treat “lung” and “kidney” asthenia syndromes describing groups of symptoms associated with respiratory and renal diseases and other disease conditions such as fatigue, asthenia after severe illness, night sweating, male and female hyposexualities, including impotence, hyperglycemia, hyperlipidemia, respiratory diseases, renal dysfunction and renal failure, liver diseases, arrhythmias and other heart diseases [13]. They have been included as a dietary supplement to maintain health and prevent disease (Appendix A Table A7) [13,135].

It has been found that most local folk/traditional healers use Caterpillar Fungus to treat 21 ailments, including erectile dysfunction, female aphrodisia, malignant tumors, bronchial asthma, bronchitis, diabetes, cough and cold, jaundice, alcoholic hepatitis, and others [21,97].

#### 2.3.2. Clinical Studies

##### 2.3.2.1. Systematic Reviews and Meta-Analyses of Randomized Clinical Trials

Several systematic reviews and meta-analyses of randomized clinical trials of *O. sinensis* in dialysis patients [136,137] with renal dysfunction [137], acute kidney injury [138], chronic kidney disease [139], diabetic kidney disease [140], lung cancer [141], chronic obstructive pulmonary disease [142,143] and in healthy human subjects [144,145] were conducted, Table 3. These meta-analyses support the efficacy and safety of *O. sinensis* for immune and respiratory health, making it a promising adjunct in both healthy and clinical populations.

An umbrella-level synthesis of these systematic reviews and meta-analyses of randomized controlled trials (RCTs) evaluating *O. sinensis* was conducted by AI assistance. The objective was to assess the strength and consistency of the clinical evidence, stratified by species and preparation type, and to grade the overall level of evidence in accordance with the European Medicines Agency (EMA) criteria for well-established use (WEU) herbal medicinal products. Evidence was synthesized narratively and comparatively, without re-pooling individual trial data, in line with accepted methodology for overviews of systematic reviews [146].

**Figure 2:**
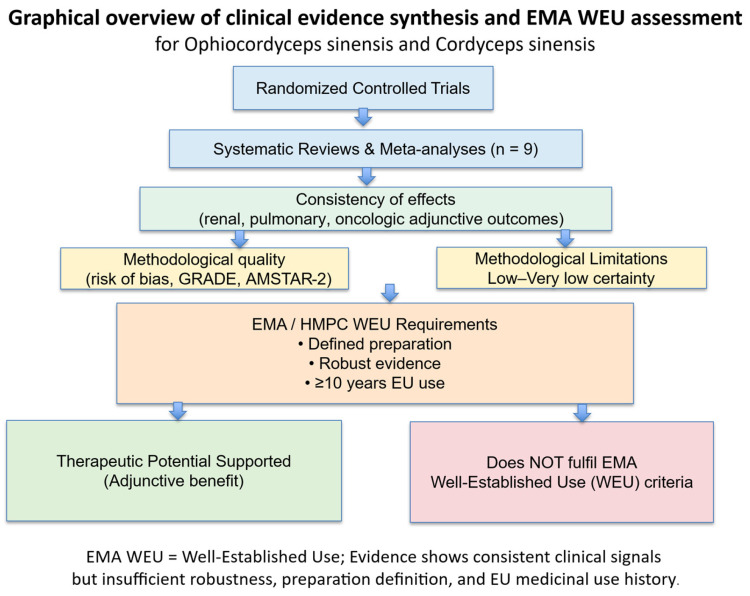
Graphical overview of clinical evidence synthesis and EMA-oriented grading of *O. sinensis*.

Table 3 summarizes the totality of clinical evidence from nine systematic reviews, stratified by species and indication, and maps the findings against the key elements required for well-established use according to EMA/HMPC principles. Figure 2 illustrates the conceptual pathway from individual randomized trials to regulatory-relevant evidence grading, highlighting the points at which the current evidence base fails to meet WEU requirements despite consistent efficacy signals.

Figure 2 summarizes the progression from randomized controlled trials through systematic reviews to regulatory-relevant evidence grading. Although consistent adjunctive clinical benefits are observed across renal, pulmonary, and oncologic indications, limitations in methodological quality, preparation heterogeneity, and lack of documented long-term EU medicinal use prevent fulfillment of EMA criteria for well-established use (WEU), which requires: (i) recognized therapeutic efficacy, (ii) acceptable safety, (iii) sufficient clinical evidence, (iv) use for at least 10 years within the European Union, and (v) a well-defined herbal substance or preparation.

Across these reviews, consistent improvements were reported in surrogate renal and inflammatory outcomes, including reductions in serum creatinine, blood urea nitrogen, C-reactive protein, and proteinuria, as well as improvements in albumin and hemoglobin levels in dialysis populations. Preventive use before contrast exposure was associated with a reduced incidence of contrast-associated acute kidney injury in several trials. However, all reviews identified substantial methodological limitations, including: (i) predominantly open-label RCTs, (ii) inadequate reporting of allocation concealment and blinding, (iii) short treatment duration and follow-up, and (iv) reliance on surrogate rather than hard clinical endpoints (e.g., mortality, progression to end-stage kidney disease). Certainty of evidence was graded as low to very low using the grading of recommendations assessment, development and evaluation (GRADE) methodology [147] in the original reviews, primarily due to risk of bias, indirectness, and imprecision.

A critical limitation for *O. sinensis* is the heterogeneity of preparations. Most clinical trials investigated fermented mycelial products (e.g., Bailing, Jinshuibao, Zhiling) rather than the wild fungus, and variability in fungal strains, manufacturing processes, and dosing regimens was observed. This heterogeneity limits the extrapolation of findings to a single, well-defined herbal substance or preparation.

Systematic reviews of *O. sinensis* evaluated its adjunctive use in COPD, lung cancer, and renal dysfunction. Across COPD studies, adjunctive use was associated with improvements in lung function parameters (e.g., FEV_1_), exercise tolerance, and quality-of-life scores. In lung cancer, meta-analyses reported improved tumor response rates, immune markers, and reduced treatment-related adverse reactions when *O. sinensis* was added to chemo- or radiotherapy. In renal dysfunction, consistent improvements in biochemical markers were observed.

Despite these positive signals, the evidence base shared several limitations:

- Trials were almost exclusively conducted in China;
- Most studies were small and short-term;
- Blinding and placebo control were largely absent;
- Outcomes were frequently surrogate or supportive rather than definitive clinical endpoints.

Accordingly, the certainty of evidence ranged from low to moderate, with significant downgrading for risk of bias and indirectness.

Many reviews used the name *O. sinensis* to describe commercial mycelial preparations that are taxonomically closer to *O. sinensis* or other related fungi. This taxonomic and pharmaceutical ambiguity represents a major obstacle for regulatory classification and evidence consolidation.

Overall, across nine systematic reviews, both *O. sinensis* preparations demonstrate biologically plausible and clinically consistent adjunctive benefits, particularly in renal disease and chronic pulmonary conditions. However, when assessed against the EMA criteria relevant to well-established herbal medicinal products, the current body of evidence does not meet the requirements for WEU, as outlined in Table 3.

This conclusion is driven not by lack of efficacy signals, but by:

- Insufficient methodological robustness;
- Absence of EU-based medicinal use documentation;
- Lack of a single, standardized herbal substance or preparation;
- Predominant reliance on surrogate outcomes.

From a scientific perspective, the evidence supports potential therapeutic value and justifies further high-quality clinical research. From an EMA regulatory perspective, the findings are more consistent with traditional herbal medicinal products rather than well-established use.

##### 2.3.2.2. Clinical Evidence for Anti-Fatigue Effects of *O. sinensis* and *C. militaris*: Implications for EMA Well-Established Use

Early placebo-controlled clinical studies examined the effects of *O. sinensis* (cordymax™ Cs-4) therapy in elderly patients with fatigue and other aging-related symptoms [148,149,150]. Compared with placebo-treated patients who showed no improvement in symptoms, most *O. sinensis* (Cs-4)-treated patients reported overall clinical improvement [149]. The subjective improvements included promotion to endurance capacity [150] and alleviation of fatigue, cold intolerance, dizziness, frequent nocturia, tinnitus, hyposexuality, and amnesia [13,149].

The results of some studies of *O. sinensis* in a limited number of healthy subject athletes suggest that it can increase exercise performance [151,152], tolerance to high-intensity exercise [153], and alleviate muscle injury [154], maintain the hemoglobin and hematocrit levels [154], while the results of a few other placebo-controlled randomized studies were not reproducible and do not show health benefits [154,155].

Two review articles summarized the effects of *O. sinensis* on aerobic performance and fitness in human studies [144,145].

Supplementation with *O. sinensis* was expected to exhibit a potential physical performance-enhancing (ergogenic) effect by increasing time to exhaustion when administered regularly for 2 to 16 weeks before exercise, although its effects on improving aerobic fitness remain inconsistent [145]. Most studies involved active, young participants; however, the ergogenic potential of *O. sinensis* in aging and sedentary populations remains poorly understood, as few studies have examined it [145].

In summary, the potential benefits of *O. sinensis* supplementation are supposed to: (i) improve aerobic performance, (ii) enhance oxygen utilization, (iii) increase maximal oxygen consumption (VO_2_max, a key indicator of aerobic fitness), and (iv) delay fatigue. However, the problems and challenges are: (i) some studies do not exhibit improvements, and the effects are inconsistent, (ii) the benefits may depend on the dosage used. Overall, while promising, more high-quality human studies are needed to fully understand its effects, especially across different populations such as older adults and sedentary individuals.

Welch et al., (2023) [144] reviewed 29 full texts of the 4308 potentially relevant articles selecting seven randomized trials of *O. sinensis* conducted with a total of 286 healthy human subjects [150,154,155,156,157,158,159,160] and applying PRISMA, the Cochrane risk-of-bias tool, the Jadad’s quality scale, and the Checklist score of Items for Reporting Trials of Chinese Herbal Medicine Formulas from CONSORT extension for Chinese herbal medicine. Formulas were applied to the data with the purpose of critically assessing the current evidence for or against the effectiveness or efficacy of *O. sinensis*. Their evaluation highlights the need to conduct high-quality, low-bias clinical trials [144]. The authors conclude that supplementation with *O. sinensis* may affect aerobic performance and could have implications for various athletic events; however, insufficient reporting of the details of the *O. sinensis* preparation used was a common issue across the included studies. Of the included studies, five reported significant pre- and post-intergroup differences in the effect of *O. sinensis* supplementation on aerobic performance [144].

Importantly, between-group analysis showed no significant changes in VO_2_max, work rate at the metabolic threshold, or work rate at the ventilatory threshold between the treatment and placebo groups [144].

In some studies, baseline (before treatment) primary outcome measures differ significantly between the placebo and *O. sinensis* groups [150], suggesting a lack of randomisation. A fatal methodological flaw in other studies was that between-group changes from baseline over time (before and after treatment) were not assessed to exclude the placebo effect. The products and their method of preparation were insufficiently characterized to demonstrate reproducibility and consistency in the results of various clinical trials.

Table A8 summarizes results of meta-analysis of randomized placebo-controlled clinical trials of *O. sinensis* in athletes [152,153,154] and healthy adults [150,158,161,162], as well as in other human subjects with symptoms of long COVID [163], mild COVID-19 [164], asthma [165], exercise fatigue [152], and *C. militaris* in mild liver dysfunction [166], depression with insomnia [167] and fatigue [153,154,161,162].

Clinical trials of *O. sinensis* include traditional extracts and cultivated mycelial products. The majority of fatigue-related trials of *O. sinensis* investigated fermented mycelial preparations, most commonly Cs-4^®^. Randomized, double-blind, placebo-controlled trials in healthy older adults demonstrated improvements in exercise tolerance, VO_2_max, ventilatory threshold, and perceived exertion, outcomes that are directly relevant to physical fatigue. Early Japanese double-blind trials demonstrated reductions in subjective fatigue scores and improved tolerance during graded exercise tests in healthy adults. Additional studies in athletes and physically active individuals reported improved exercise tolerance and oxygen-related biomarkers, although findings were inconsistent across studies. Null results were frequently observed in highly trained populations, suggesting a limited ergogenic ceiling effect and reinforcing the notion that fatigue-modulating effects may be most relevant in sub-optimally conditioned or aging individuals.

This section critically reviews fatigue-specific clinical trials of *O. sinensis* in healthy adults, older adults, and individuals with post-COVID-19 conditions. It assesses the strength of the evidence against the European Medicines Agency (EMA) criteria for well-established use (WEU). EMA WEU requirements include recognized efficacy, acceptable safety, consistent clinical evidence, a well-defined herbal preparation, and at least 10 years of medicinal use within the European Union. Species are analyzed separately due to regulatory and pharmacognostic relevance.

Appendix A Table A9 is focused exclusively on long COVID/post-COVID fatigue. As summarized in the table, direct clinical evidence for the use of *O. sinensis* in long COVID-related fatigue is currently limited but emerging. A recent randomized, waitlist-controlled trial in patients with long COVID demonstrated that Cs-4^®^ significantly improved fatigue severity, functional capacity, and health-related quality of life compared with usual care [163]. This study represents the most direct clinical evidence for an anti-fatigue effect of *O. sinensis* in a post-viral population. However, the trial was limited by regional recruitment, short follow-up, and reliance on patient-reported outcomes. Evidence on COVID-19-related fatigue is emerging but remains limited. An industry-sponsored randomized study reported improvements in fatigue and recovery time during acute COVID-19 when *O. sinensis* capsules were used as add-on therapy. However, the lack of peer review and methodological transparency substantially reduces the evidentiary weight.

Fatigue-specific clinical trials of *O. sinensis* demonstrate biologically plausible and clinically observable benefits, particularly in aging and post-viral populations. However, when evaluated against EMA/HMPC standards, the evidence base remains insufficient for WEU designation, primarily due to methodological limitations and heterogeneity in preparation.

Overall, across healthy, aging, and post-infectious populations, *O. sinensis* demonstrates biologically plausible and clinically observable anti-fatigue effects, with the strongest signals seen in older adults and long COVID patients. However, under EMA/HMPC standards, the current evidence base is insufficient to support well-established use due to methodological limitations, evidence quality (most trials are small, short-term, and use heterogeneous or surrogate fatigue endpoints); GRADE certainty would be low to moderate: downgrading was primarily due to risk of bias (blinding, allocation concealment), inconsistency across populations, indirectness (exercise performance vs. validated fatigue scales), and imprecision (small sample sizes), preparation heterogeneity (fermented mycelium, different strains, combination products), preventing the definition of a single herbal preparation, and a lack of documented long-term EU medicinal use. From a scientific perspective, the evidence justifies further high-quality trials using validated fatigue endpoints. From a regulatory perspective, the data align more closely with traditional use of herbal medicinal products than with well-established use (WEU) status, and emerging clinical signals warrant further confirmatory trials using validated fatigue instruments. Future studies should prioritize validated fatigue scales, longer follow-up, and standardized preparations to strengthen both scientific and regulatory credibility.

### 2.4. Systems-Level Mechanisms of O. sinensis, C. militaris, and Cordycepin: An Integrative Network Pharmacology and Experimental Evidence Review

Caterpillar Fungus and Orange Cordyceps are medicinal fungi traditionally valued for their restorative, endurance-enhancing, and homeostasis-supporting properties. These attributes align with the modern concept of adaptogenic activity, characterized by improved resistance to physical, chemical, and biological stressors [26,28,134].

Both fungi contain diverse bioactive metabolites, including nucleosides (adenosine, 3-deoxy-adenosine/cordycepin), sterols, peptides, and polysaccharides. The pharmacological breadth of *O. sinensis* suggests multi-target effects rather than classical single-receptor drug actions. Network pharmacology provides a systematic approach to deciphering these complex interactions, integrating predicted targets, signaling pathways, molecular docking, and multi-omics data.

Network pharmacology, combining computational target prediction, pathway enrichment, and systems biology, has become a significant framework for elucidating the complex multi-target actions of such herbal [168,169,170,171,172,173], including *O. sinensis*, *C. militaris*, and cordicepin and other fungi preparations [68,174,175]. Many network pharmacology studies of *O. sinensis* employ a so-called “reverse pharmacology” approach [176], based on plants described in ancient texts or on the empirical knowledge of traditional healers, and are focused on elucidating their mechanisms of action, which is typical of TCM. However, most reports remain scattered, disease-specific, or lacking integration with wet-lab validation. Despite numerous studies [57,60,103,140,144,174,175,177,178,179,180,181,182,183,184,185,186,187,188,189,190,191,192,193,194,195,196,197,198,199,200,201,202,203,204,205,206,207,208], no comprehensive synthesis has unified the mechanistic insights across species, compounds, and disease contexts. A unified synthesis is needed to understand the core pharmacological principles of *O. sinensis* and *C. militaris*.

This section provides the most comprehensive overview to date of network pharmacology analyses of *O. sinensis*, *C. militaris*, and cordycepin, integrating computational predictions and experimental evidence to derive a consolidated mechanistic model. This study integrates 37 network pharmacology investigations, including 22 experimental validation studies, along with Appendix A Table A10 and Table A11 [177,178,179,180,181,182,183,184,185,186,187,188,189,190,191,192,193,194,195,196,197,198,199,200,201,202,203,204,205,206], to define the adaptogenic potential and systems-level pharmacology of Caterpillar Fungus, Orange Cordyceps, and cordycepin.

Across the studies, the workflow is similar: selection of active constituents of Cordyceps → predict molecular targets → build protein–protein interaction/pathway networks → dock key compounds to target proteins → sometimes validate in cells/animals. Importantly, these are hypothesis-generating in silico, so their strength depends on follow-up experiments (Appendix A Table A10 and Table A11).

**Figure 3:**
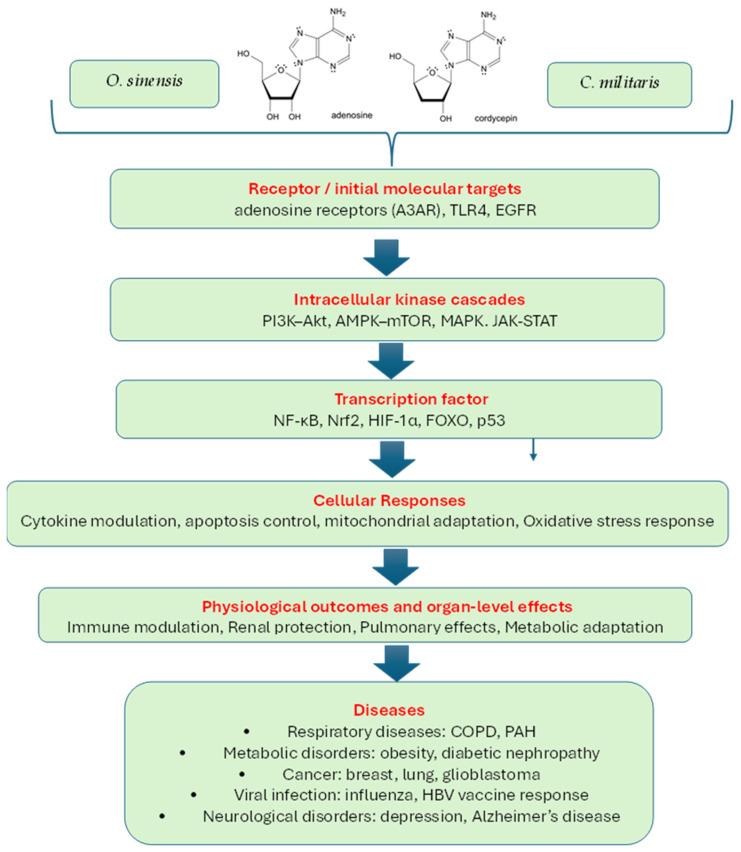
Hierarchical mechanistic integration of network-derived targets of *O. sinensis*, adenosine, *C. militaris*, and cordycepin-induced adaptive stress response, network signaling pathways, and biological functions, which are summarized in Appendix A Table A13, including primary function related to: (i) PI3K–AKT/FOXO–SIRT metabolic survival axis, (ii) AMPK–mTOR–autophagy axis, (iii) NRF2–KEAP1 antioxidant axis, (iv) TLR4–MyD88–NF-κB/MAPK inflammatory axis, (v) NLRP3 Inflammasome/Pyroptosis, and (vi) HIF-1 hypoxia response. *O. sinensis*, adenosine, *C. militaris*, and cordycepin act as multi-target modulators converging on integrated adaptive stress-response signaling, immunometabolic, and mitochondrial pathways.

Across fungal species and study designs, highly consistent mechanistic hubs emerged: inflammatory cytokines (TNF, IL-6, IL-1β), stress-activated kinases (MAPK1/3/8), survival pathways (AKT1), mitochondrial regulators (FOXO3, HIF-1), apoptotic mediators (CASP3, BAX/BCL2), transcriptional co-regulators (CREBBP, EP300, FOXO3), and metabolic enzymes (IDH1, CYP19A1), Figure 3, Table A12 and Table A13.

The most frequently enriched recurrent pathways included PI3K–Akt, MAPK, NF-κB, apoptosis, oxidative-stress regulation, and AMPK–SIRT1–PGC-1α, the latter closely linked to metabolic and mitochondrial adaptation, Appendix A Table A12. This convergence indicates that *O. sinensis* and *C. militaris* metabolites act not through a single canonical receptor but through a broad network modulation that affects immunity, metabolism, mitochondrial function, and cell-survival signaling—consistent with the systems-level pharmacology expected of adaptogenic botanicals.

Experimental evidence validated predicted mechanisms in obesity, COPD, pulmonary hypertension, cancer, influenza, and vaccine immunogenicity (Appendix A Table A10), Figure 3.

Experimental validation studies evidence confirmed network predictions in:

- Immune modulation via TLR4/TNF-α [182];
- Cancer apoptosis via PI3K–Akt and caspase regulation [187];
- Apoptotic pathway activation in cancer [178,187];
- Cytokine normalization and lung restoration in COPD [174];
- PAH through apoptosis and vascular remodeling repair [191];
- Obesity/metabolic syndrome via AKT1/MAPK14/GSK3B [192];
- HBV vaccine response enhancement [177].

The integrative analysis of network pharmacology data combined with experimentally validated studies demonstrates that *O. sinensis*, *C. militaris*, and cordycepin share a convergent mechanistic architecture centered on immune modulation, stress-kinase regulation, metabolic adaptation, mitochondrial support, and apoptosis control. The repeated identification and validation of PI3K–Akt, MAPK, NF-κB, and AMPK–SIRT1–PGC-1α pathways indicate that *O. sinensis*, *C. militaris*, and cordycepin are systems-level adaptogenic agents capable of normalizing physiological functions across multiple organ systems. These findings support the therapeutic potential of standardized preparations and justify further translational research, particularly in immunometabolic and chronic inflammatory diseases.

Table 4 presents the key findings of network pharmacology studies and the characteristic differences between *O. sinensis* [139,197,198,208] and *C. militaris* preparations [182,183,185,206] (Appendix A Table A10 and Table A11).

The implications and predictions for health claims inferred from the network pharmacology studies, Table 4, can be summarized as follows:

- *O. sinensis* exhibits broad-spectrum organ support and multi-pathway modulation, especially in chronic diseases and viral infections. The most defensible, network-anchored claims are around kidney support/adjunct in chronic kidney disease, inflammation/oxidative stress, and respiratory immune modulation—with some clinical meta-analytic support but still needing higher-quality trials [136,139].
- *C. militaris* shows targeted cytotoxicity and immune activation, making it more suitable for oncology and immunotherapy applications. *C. militaris* preparations, explicitly characterized by high content of cordycepin, have the strongest network-mechanistic case for anticancer mechanisms, immune modulation (TLR4–TNF, macrophage polarization), and metabolic/urate axes. The evidence is growing, but disease-specific clinical endpoints remain limited [182,183,185].

Pathways are stratified according to an evidence-tier framework:

- Tier 1 (Prediction Only): Identified through in silico network modeling and enrichment analysis without biological confirmation.
- Tier 2 (Experimental Validation): Supported by in vitro or in vivo mechanistic studies.
- Tier 3 (Clinical Alignment): Mechanistic pathways supported by human clinical biomarker modulation or RCT endpoints.

This structured presentation reduces target inflation bias and visually distinguishes hypothesis-generating predictions from experimentally or clinically supported mechanisms.

Convergent evidence from network pharmacology, experimental studies, and multi-omics analyses demonstrates that *O. sinensis*, *C. militaris*, and cordycepin share a unified mechanistic architecture centered on immune modulation, stress-kinase regulation, mitochondrial adaptation, and metabolic homeostasis. This integrated systems pharmacology supports their classification as adaptogenic natural products with relevance to chronic inflammatory, metabolic, and immunological diseases. Future work prioritizing standardized preparations and mechanistically powered clinical studies will be essential for therapeutic translation.

**Figure 4:**
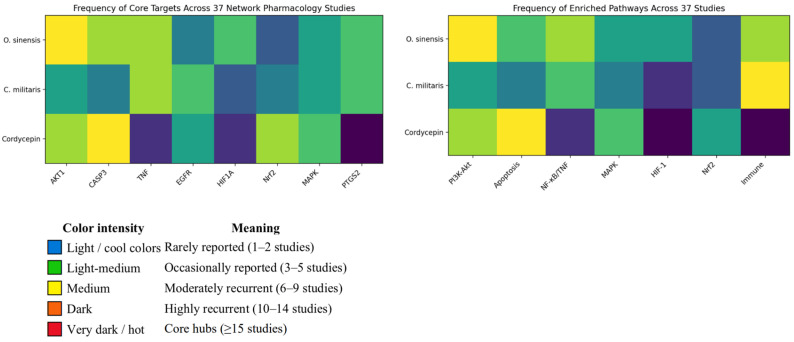
The heatmaps are frequency heatmaps, not expression heatmaps. They visualize how often a given target or pathway appears across your 37 network pharmacology studies, separated by: *O. sinensis*, *C. militaris*, and cordycepin. Each cell indicates the number of independent publications reporting a given target or pathway. Highly frequent targets and pathways may reflect research prevalence and database structure rather than relative biological importance. Color intensity reflects convergence level: (i) warmer/darker colors = higher recurrence across studies (high-confidence hubs), (ii) cooler/lighter colors = lower recurrence (hypothesis-generating pathways). Functional modules are grouped into: (i) Immunoinflammatory signaling (e.g., NF-κB, TNF, TLR pathways), (ii) metabolic and mitochondrial regulation (e.g., AMPK–mTOR, SIRT1–PGC-1α), (iii) apoptosis and cell-survival signaling (e.g., PI3K–Akt, CASP3), (iv) oxidative stress response (e.g., Nrf2–KEAP1 axis).

Table A14 presents an evidence-tier framework that distinguishes Tier 1 (in silico prediction), Tier 2 (experimental validation), and Tier 3 (clinical alignment). Figure 4 and Table 4 reflect validation levels and reduce potential target inflation bias.

Figure 4 presents frequency heatmaps summarizing the recurrence of predicted and validated targets and enriched pathways across 37 independent network pharmacology studies involving *O. sinensis*, *C. militaris*, and cordycepin. Heatmaps were generated using AI to visualize the frequency of reported targets and enriched pathways across 37 network pharmacology studies. For each entity (*O. sinensis*, *C. militaris*, cordycepin), the number of independent publications reporting a given target or pathway was counted. Color intensity represents recurrence frequency, with warmer colors indicating higher convergence across studies.

Table 5 shows the effects of cordycepin on adaptive stress–response signaling pathways and physiological roles [36,209,210,211,212,213,214,215,216,217]. Appendix A Table A13 presents the major adaptive stress response pathways modulated by cordycepin, highlighting key genes, their biological roles, and the primary supporting studies.

Caterpillar Fungus, Orange Cordyceps, and 3-deoxyadenosine exhibit a unified adaptogenic pharmacology that involves immune modulation, mitochondrial enhancement, stress-signal regulation, and metabolic homeostasis. These effects arise through multi-target synergy across PI3K–Akt, MAPK, NF-κB, apoptosis, and AMPK–SIRT1–PGC-1α pathways. The strong convergence of network and experimental evidence supports their potential in immunometabolic and stress-related disorders.

Our findings align with a recent review providing evidence-based rationale for Chinese traditional medicinal mushrooms, which demonstrate multi-target anti-inflammatory activity by modulating key cellular mediators (macrophages, regulatory T cells, natural killer cells) and signaling pathways (NF-κB, MAPK, NLRP3 inflammasome, Nrf2/HO-1) of mushrooms for chronic inflammation management [68].

Table 6 summarizes key genes involved in cordycepin-induced adaptive stress–response signaling pathways and their physiological roles [33,34,35,36,37,38].

### 2.5. Food and Drug Regulation of O. sinensis and C. militaris in Western and Oriental Worlds

Wild *O. sinensis* has medicinal rather than culinary use in TCM, traditionally as a tonic, and is not classified as an edible mushroom in food codes. Cultured mycelium of *O. sinensis* can be food-grade or supplement-grade, depending on the strain and national registration (Appendix A Table A15). *C. militaris*, by contrast, is generally recognized as edible and is widely cultivated as a functional food mushroom. Table 7 summarizes the regulatory/edible/novel-food status of *O. sinensis* vs. *C. militaris* across major regions, with key references to support claims. Data derived from peer-reviewed studies [16,218], official food and drug regulatory websites, and Rapid Alert System for Food and Feed (RASFF) notifications [219,220,221,222,223,224,225,226,227,228].

In China, *C. militaris* has a new resource-food status (since 2009) [218], so it is widely used in domestic foods/health foods. In the Republic of Korea and Japan, *C. militaris* is regarded as edible/medicinal under functional food or Kampo frameworks, whereas *O. sinensis* is used as a medicinal fungus rather than a culinary mushroom. In the USA, both are typically sold as dietary supplements, avoiding disease claims to stay out of the “new drug” category. Overall, in the EU, *O. sinensis* (mycelium and fruiting body) is used as a food supplement without a novel food authorisation (other food uses may still be novel). *C. militaris* (mycelium and fruiting body) is novel and not yet authorized; placing supplements with *C. militaris* on the EU market requires a successful novel food authorisation or an exemption that does not currently exist and can be expected at the Rapid Alert System for Food and Feed (RASFF) alerts portal, see Appendix A [225,226,227,228], Table A15. The Appendix A Table A16, Table A17, Table A18, Table A19 and Table A20 show characteristic features of *O. sinensis* and *C. militaris* across key pharmacopeias and regulatory frameworks, including health claims and medicinal uses.

### 2.6. Resilience Biology, Inflammation, and Adaptogens: An Umbrella Review of Biological Evidence and Regulatory Frameworks Across Europe, the United States, and Asia

Stress responses and inflammation are evolutionarily conserved adaptive processes essential for survival and recovery, restoring homeostasis following injury or challenge [229,230,231,232,233,234,235,236], as shown in Appendix A Table A21. While inflammation is firmly established as a pathological and regulatory target in Western medicine [237,238], stress adaptation and resilience biology remain poorly integrated into regulatory frameworks, particularly within the European Union [239,240,241]. Adaptogens are proposed to enhance adaptive capacity and nonspecific recovery in various diseases and disorders [26,28,134,242]. Despite accumulating biological evidence, adaptogens lack regulatory recognition in the EU [241], while receiving broader acceptance in the United States [237,243], and Asia [244].

This umbrella review section synthesizes evidence from systematic reviews, narrative reviews, and regulatory documents to compare (i) biological definitions of inflammation, stress, and adaptive stress responses; (ii) their physiological roles and resolution mechanisms; and (iii) regulatory acceptance of adaptogenic botanicals across the European Union (EU), United States (US), and selected Asian jurisdictions. A structured umbrella review approach was applied to peer-reviewed reviews (2000–2024) addressing inflammation, stress physiology, adaptogens, and regulatory science. Regulatory guidance from EFSA, EMA, FDA, and Asian authorities was examined. Findings were narratively synthesized.

Stress and inflammation share common adaptive purposes and resolution dynamics (Table 8). Both stress and inflammation are beneficial in acute settings and harmful in chronic settings (Appendix A Table A20). Chronic stress drives chronic inflammation, and chronic inflammation feeds back into stress circuits.

Unfortunately, terms such as stress, fatigue, and adaptation have not been formally recognized as a regulatable pathological entity in the EU. The reason lies in the basic concepts of health and fatigue in Western and Oriental medical paradigms, as reflected in the food and drug regulatory authorities in Europe, the USA, China, India, and Japan. Key conflict: traditional systems intervene upstream of disease, whereas EU drug regulators say “no disease exists yet” (Table 9 and Table 10).

Why is the “anti-inflammatory” effect a recognized regulatory term, but “antistress”, “anti-fatigue”, and “stress-protective” are not recognized in Europe? Regulatory reasoning is that: inflammation is a defined pathological process, established clinical biomarkers exist, precise pharmacological mechanisms are known, and historical drug precedents exist (NSAIDs, corticosteroids). Consequently, the European Medicines Agency accepts anti-inflammatory mechanism-based claims and botanical medicines with demonstrated effects on inflammatory mediators.

Meanwhile, the terms “antistress”, “anti-fatigue”, and “stress-protective” are not accepted by EMA because of regulatory concerns, including: (i) “Stress” is considered a normal life experience, not a disease, (ii) claims imply psychological or psychiatric effects, and (iii) risk of unsubstantiated or exaggerated health claims. Scientific challenges include: (i) stress is considered contextual and subjective, (ii) no single, validated disease definition, and (iii) biomarkers are indirect, variable, and situation-dependent. Thus, authorities avoid approving terms that: (i) lack precise physiological targets, (ii) cannot be consistently quantified, and (iii) blur boundaries between food, supplements, and medicines.

A core regulatory paradox is that vitamins and antioxidants are recognized, but adaptogens are not. The reason is that EU Regulation favors selected target, single-pathway, disease-linked, measurable mechanisms, while adaptogenic effects are systems-level and contextual. While adaptogens aim to normalize stress responses rather than inhibit a specific pathological pathway, this does not fit the reductionist regulatory model.

EU drug authorities consider stress as an adaptive life process, but not a disease target, and adaptogens as a regulatory misfit, but not a scientific invalidity. Adaptogens fail not scientifically, but structurally. EU drugs regulators lack a legal category for resilience enhancers. Adaptogens challenge regulatory frameworks because they support resilience rather than treat disease.

In 2007, EMEA’s “Reflection paper on the adaptogens concept” clarified that the term “adaptogen” is applied to different herbal medicinal products (including preparations derived from Eleutherococcus, Ginseng, and Rhodiola) that have the capacity to normalize bodily functions and strengthen systems compromised by stress [241]. They are reported to have a protective effect on health against a wide variety of environmental assaults and emotional conditions [241]. EMA approved EU Community Monographs for Rhodiola, Eleutherococcus, and Ginseng as traditional herbal medicinal products for the relief of stress-related symptoms, such as fatigue and exhaustion [245]. EMEA “Reflection paper on the adaptogens concept” concluded “*The concept of adaptogens is sufficient to be considered in the assessment of traditional herbal medicinal products (e.g., monograph on Eleutherococcus root). As the term “adaptogen” is considered not appropriate for a marketing authorisation, more clinical studies, and data on the efficacy in a well-defined clinical condition would be necessary*” [241]. When the second sentence is taken out of context from the EMA conclusion, e.g., on Wikipedia, it creates a misleading impression of the scientific validity of the adaptogenic concept. Over the last two decades, our understanding of the mechanisms of action and clinical conditions of adaptogens has been substantially enriched through network pharmacology and molecular biology studies [26,28,134,171,173,246,247], whereas the clinical evidence of their efficacy remains insufficient due to various methodological shortcomings and limited characterization of study products [241].

Adaptogens are rejected in the EU not because they lack biological validity, but because European regulation has no legal concept of resilience. In contrast, Asia is built on it, and the US pragmatically tolerates it (Table 10).

Adaptogens demonstrate modulatory effects on immune, inflammatory, neuroendocrine, and metabolic pathways consistent with resilience biology [26,28,134,242,246] yet lack a regulatory category in Europe. The US adopts a permissive structure–function approach, while Asian systems explicitly integrate adaptogens within traditional and functional medicine paradigms.

This review clarifies conceptual and regulatory barriers to recognizing resilience-supporting interventions and informs future regulatory innovation. Adaptogens are not rejected due to insufficient biology, but because current regulatory frameworks lack concepts for resilience and adaptive capacity. Establishing a regulatory category for resilience-supporting physiological modulators could align regulation with contemporary systems biology.

## 3. Discussion

### 3.1. An Integrative Network Pharmacology and Systems-Level Mechanisms of Ophiocordyceps sinensis, Cordyceps militaris, and Cordycepin

The quantitative synthesis of 37 network pharmacology studies reveals a highly convergent and biologically coherent systems-level pharmacological architecture underlying the activities of *O. sinensis*, *C. militaris*, and cordycepin. Rather than supporting isolated, disease-specific mechanisms, aggregated networks consistently converge on a limited set of high-centrality molecular hubs and signaling modules that govern cellular stress adaptation, immunometabolic homeostasis, inflammatory tone, and survival–death decisions.

#### 3.1.1. Convergence on a Conserved Stress-Adaptive Signaling Core

Across species and preparations, the most frequently identified hubs—including AKT1, CASP3, TNF/NF-κB, MAPKs, HIF-1α, and Nrf2—define a conserved stress-adaptation core network. These nodes integrate metabolic sensing (PI3K–Akt, AMPK–mTOR), redox regulation (Nrf2), inflammatory control (TNF/NF-κB, TLR signaling), and apoptotic regulation (caspase cascades), Appendix A Table A20. The repeated emergence of this hub constellation across heterogeneous disease models indicates that *O. sinensis* and *C. militaris*-derived products function as coordinated regulators of cellular resilience systems rather than as single-target agents.

This convergence provides a mechanistic foundation for the traditional classification of Cordyceps mushroom as tonic or adaptogenic medicines and is consistent with contemporary network-medicine models, in which therapeutic benefit arises from distributed modulation of interacting stress-response circuits rather than maximal perturbation of isolated molecular targets.

#### 3.1.2. Species-Level Pharmacology Reflects Network Breadth, Not Redundancy

Although *O. sinensis* and *C. militaris* share substantial overlaps in their predicted and validated networks, their frequency profiles reveal distinct pharmacological emphases. *O. sinensis* shows preferential enrichment in immune–pulmonary–renal metabolic networks, with frequent involvement of HIF-1, AGE–RAGE, VEGF, chemokine, and inflammatory lipid pathways. This pattern suggests dominant engagement of hypoxia adaptation, vascular regulation, tissue microenvironment remodeling, and chronic inflammatory control, aligning closely with its traditional and contemporary investigation in respiratory, renal, ischemic, and fatigue-associated disorders.

In contrast, *C. militaris* demonstrates stronger relative representation of immune receptor signaling, oxidative-stress pathways, and metabolic enzymes, consistent with a mechanistic orientation toward immune modulation, antiviral and anticancer responses, and metabolic regulation. The prominence of TLR-associated and lipid-inflammatory pathways supports its emerging positioning as an immune-functional and anticancer medicinal fungus.

Thus, while the two species occupy overlapping regions of pharmacological network space, they exhibit distinct systems-level biases rather than functional redundancy.

#### 3.1.3. Cordycepin Acts as a Dominant Molecular Effector Within Broader Fungal Networks

Cordycepin-centered networks display a distinct topological signature, characterized by high-centrality intracellular signaling hubs that control apoptosis, kinase cascades, and metabolic checkpoints. Compared with whole-fungus preparations, cordycepin exhibits a disproportionately strong action of caspase signaling, AMPK–mTOR, PI3K–Akt, Wnt/β-catenin, and Nrf2 pathways, indicating a more targeted regulation of intracellular stress integration, metabolic reprogramming, and survival–death switching.

These patterns support a model in which cordycepin acts as a dominant molecular effector that drives a subset of *C. militaris* pharmacology. However, the broader immunological, vascular, and lipid–steroid signaling observed for whole-fungus preparations likely reflects synergistic contributions from additional nucleosides, polysaccharides, sterols, peptides, and secondary metabolites. Consequently, cordycepin recapitulates but does not fully reproduce the systems-level pharmacology of *C. militaris*.

#### 3.1.4. Validated Versus Predicted Networks Define Evidence Tiers

Stratification of validated and prediction-only studies demonstrates that the most frequently recurring hubs and pathways—PI3K–Akt, NF-κB/TNF, apoptosis, MAPKs, HIF-1, Nrf2, and immune signaling—are robust to evidence filtering and consistently supported by experimental data. In contrast, lower-frequency pathways are concentrated in prediction-only studies, representing hypothesis-generating extensions rather than confirmed pharmacological cores.

This separation establishes an evidence-tier framework that may guide translational prioritization: validated networks provide mechanistic foundations suitable for biomarker development and clinical positioning, whereas prediction-only networks identify directions for targeted experimental expansion.

#### 3.1.5. Implications for Natural-Product Systems Pharmacology

Collectively, these findings position Caterpillar Fungus, Orange Cordyceps-derived products as archetypal systems-level natural therapeutics whose biological activities arise from coordinated regulation of adaptive stress-response networks. The consistent involvement of immunometabolic, redox, hypoxia, and apoptosis modules underscores their potential relevance not only for disease intervention but also for functional resilience, recovery support, and maintenance of physiological function during aging.

Moreover, the alignment between traditional tonic indications and modern network-level convergence highlights network pharmacology as a translational bridge between ethnopharmacology and molecular systems medicine, enabling rational development, standardization, and positioning of medicinal fungi.

The integrative analysis of network pharmacology data, combined with experimentally validated studies, demonstrates that Caterpillar Fungus, Orange Cordyceps, and cordycepin share a convergent mechanistic architecture centered on immune modulation, stress-kinase regulation, metabolic adaptation, mitochondrial support, and apoptosis control. The repeated identification and validation of PI3K–Akt, MAPK, NF-κB, and AMPK–SIRT1–PGC-1α pathways indicate that Orange Cordyceps acts as a systems-level adaptogenic agent, capable of normalizing physiological functions across multiple organ systems. These findings support the therapeutic potential of standardized preparations of Caterpillar Fungus and Orange Cordyceps and justify further translational research, particularly in immunometabolic and chronic inflammatory diseases.

The striking convergence across species, compounds, and diseases indicates that Caterpillar Fungus, Orange Cordyceps, and cordicepin operate as network-level adaptogens. Their actions modulate: immune homeostasis, mitochondrial resilience, redox balance, stress-kinase signaling, and metabolic efficiency. These domains correspond to the core characteristics of adaptogenic botanicals.

Although 3-deoxyadenosine undergoes rapid metabolism and has low plasma concentrations, these levels are compatible with hormetic activation of AMPK, SIRT1, and FOXO pathways—mechanisms central to metabolic and mitochondrial adaptation.

Alignment of network predictions with experimental data across diverse disease models strongly supports the robustness of the mechanistic model.

#### 3.1.6. Limitations

Limitations of these studies include heterogeneity of extracts, target inflation bias, uneven experimental validation, and overrepresentation of cancer/inflammation models. Variability in fungal strains, extraction methods, and analytical techniques remains a challenge. More standardized preparations and human mechanistic trials are required.

**Figure A2:**
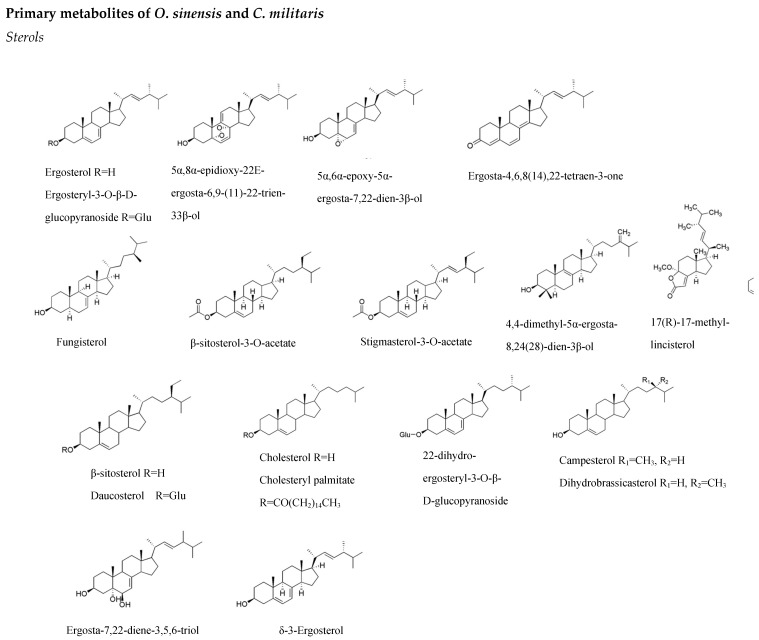
Primary metabolites: sterols.

**Figure A3:**
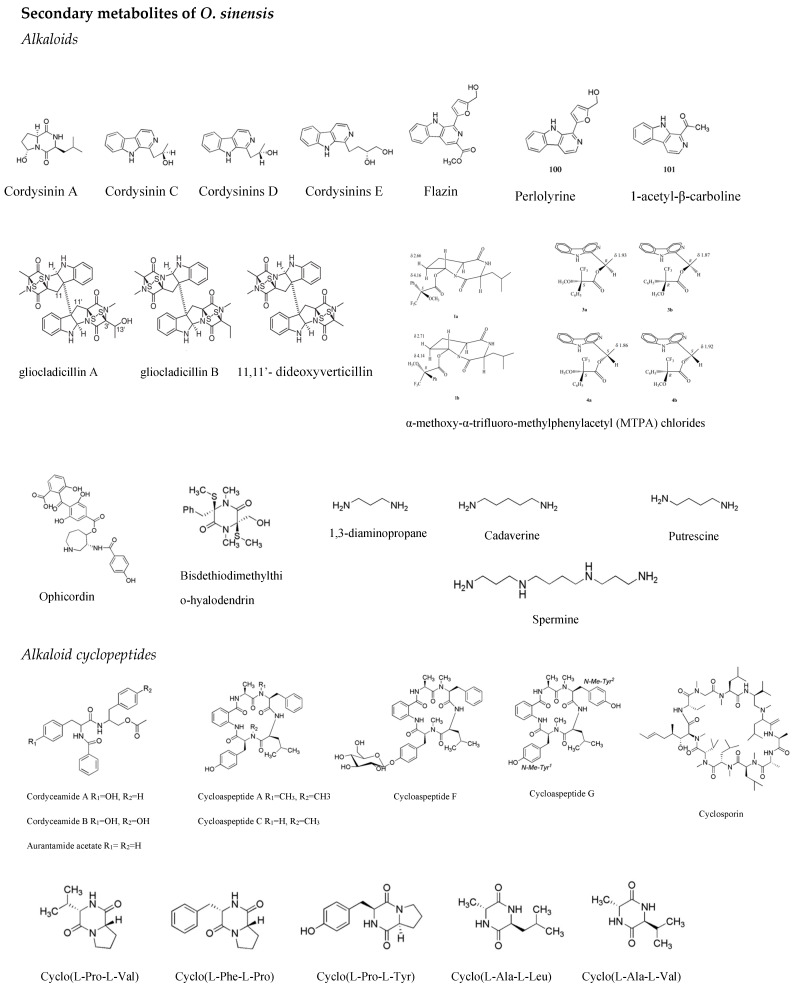
Secondary metabolites of *O. sinensis*.

**Figure A4:**
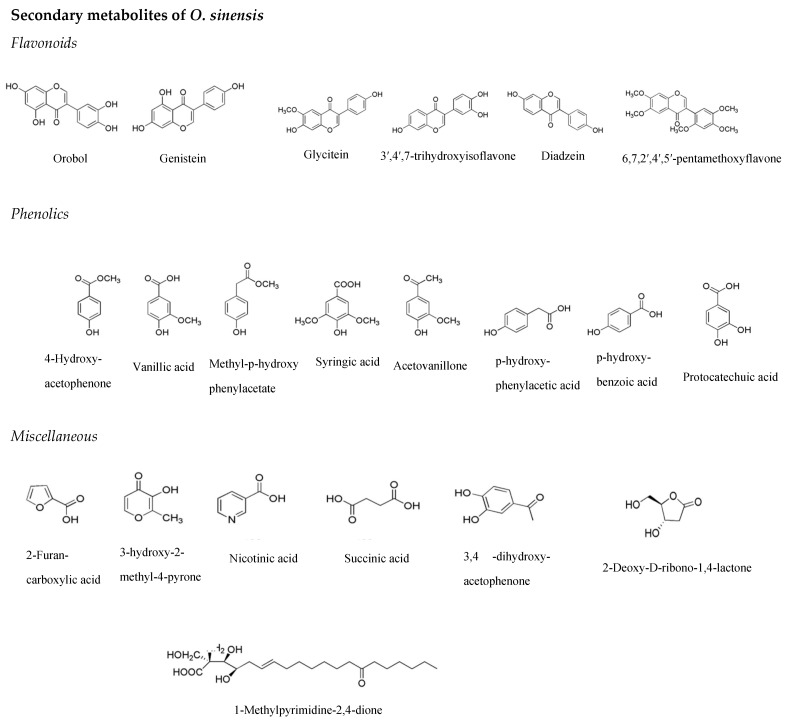
Secondary metabolites of O. sinensis (continued).

**Figure A5:**
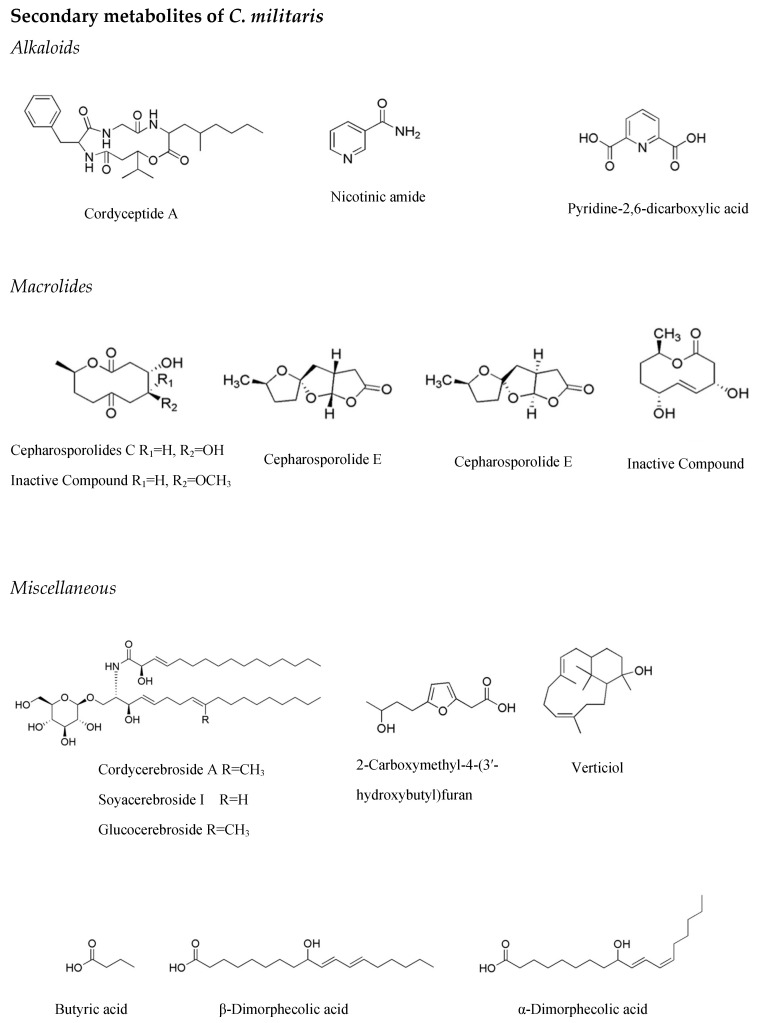
Secondary metabolites of *C. militaris*.

As a matter of fact, all the network pharmacology studies of *O. sinensis* were conducted on a limited number of one (cordicepin) to 13 bioactive constituents/compounds (Table A10 and Table A11) in the total of 84 compounds identified in *O. sinensis*, Figure A1, Figure A2, Figure A3, Figure A4 and Figure A5, and Table A3. Many other bioactive fungi secondary metabolites (Table A3, Table A4, Table A5 and Table A6), specifically alkaloids, flavonoids, sterols, cyclodipeptides, and other bioactive *O. sinensis* secondary metabolites, were not included in the network analysis. That is an essential omission that may affect the outcomes of these studies, and the overall evidence supporting health claims, and medical use in drug authorities’ assessments. Most network pharmacology studies analyze only a subset of fungal metabolites because compound selection is typically based on overlap between known molecular targets and disease-associated targets. Consequently, many primary and secondary metabolites listed in Section 2.1 remain outside current network analyses because their targets are unknown. The proposed core network in these studies reflects the subset of metabolites currently under study rather than responses to the full chemical diversity of *O. sinensis* and *C. militaris*. The lack of knowledge can be overcome in future studies using various molecular biology methodologies, including transcriptome-wide microarray profiling of gene expression experiments on extracts containing all extractive constituents of *O. sinensis* or *C. militaris*, in a non-targeted study design discussed in Section 3.1.7.

#### 3.1.7. Future Perspectives

Network pharmacology is a relatively new, biology-based interdisciplinary field that combines principles of pharmacology and systems biology to study the complex interactions between drugs, their targets (e.g., receptors or enzymes), pathological processes, and diseases within integrated, holistic systems, exploring the polypharmacology and toxicology of drugs by utilizing the computational tools and network analysis algorithms.

Depending on the aims, the design and methodology of NP studies can be specific disease-targeted or non-targeted studies, where

- Disease-targeted bioinformatic-based network analysis reveals the molecular mechanisms of action, common molecular targets of active compounds, and stimulus-response coupling signaling pathways [57,60,103,140,144,174,175,177,178,179,180,181,182,183,184,185,186,187,188,189,190,191,192,193,194,195,196,197,198,199,200,201,202,203,204,205,206,207,208]. Such study design and methodology do not account for synergistic and antagonistic interactions in networks induced by various constituents of the complex, multi-component extracts, which can lead to unexpected outcomes, e.g., [246,247].
- Non-targeted transcriptome-wide microarray profiling of gene expression-based experiments following integrated metabolomics and network analysis, revealing all molecular targets of active compounds and their response, coupling signaling pathways. The results of these studies can predict unknown physiological functions, health consequences, and therapeutic indications due to synergistic and antagonistic interactions [171,173,246,247].
- Currently, all conducted network pharmacology studies on Caterpillar Fungus, Orange Cordyceps, and cordicepin are limited to disease-targeted investigations of Chinese TCM prescriptions for the treatment of kidney diseases and related disorders. Non-targeted transcriptome-wide microarray profiling of gene expression-based experiments can lead to the discovery of new therapeutic indications and functional claims.

All published network pharmacology studies of *O. sinensis* have a targeted design focused on a specific disease. The search for unknown therapeutic uses can be pursued by exploring alternative study designs based on gene expression, and by using transcriptomics, proteomics, and metabolomics (omics-wide array) methodologies to discover new targets associated with potential pathologies and diseases.

Further perspectives include integration of multi-omics, AI-driven causal inference, pharmacokinetic–network modeling, and biomarker-guided trials.

### 3.2. Nucleosides Adenosine and Cordycepin as Mediators of Adaptive Stress Response and Anti-Fatigue Activity of O. sinensis, and C. militaris

**Figure A6:**
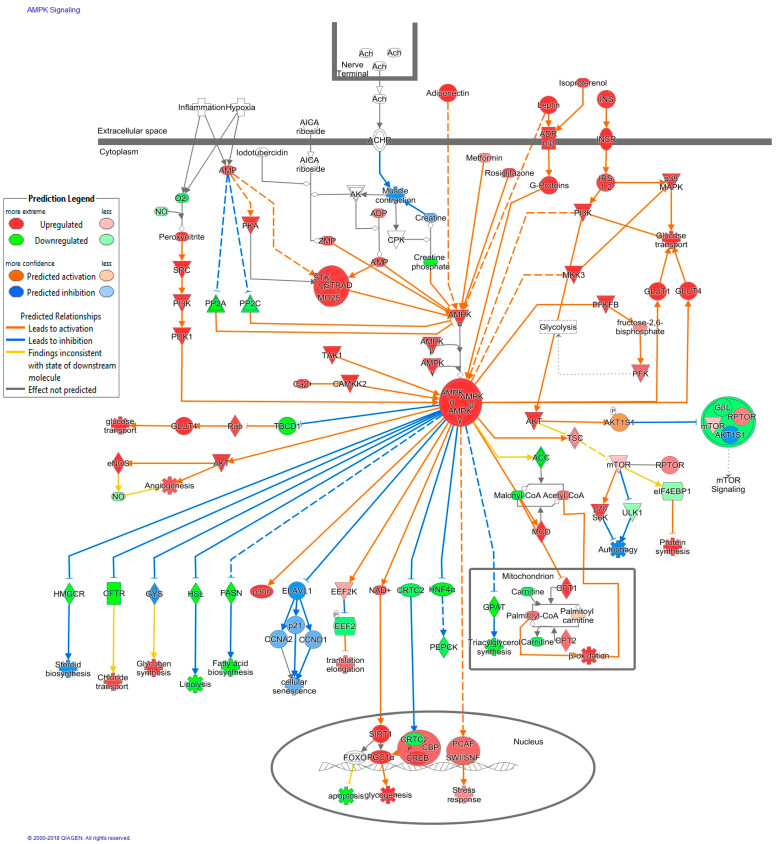
Activation of the AMPK signaling pathways and multiple effects on stress response, cellular senescence, apoptosis, autophagy, angiogenesis, protein and glycogen synthesis, steroids biosynthesis, mTOR signaling, and other canonical adaptive signaling pathways [246].

**Figure A7:**
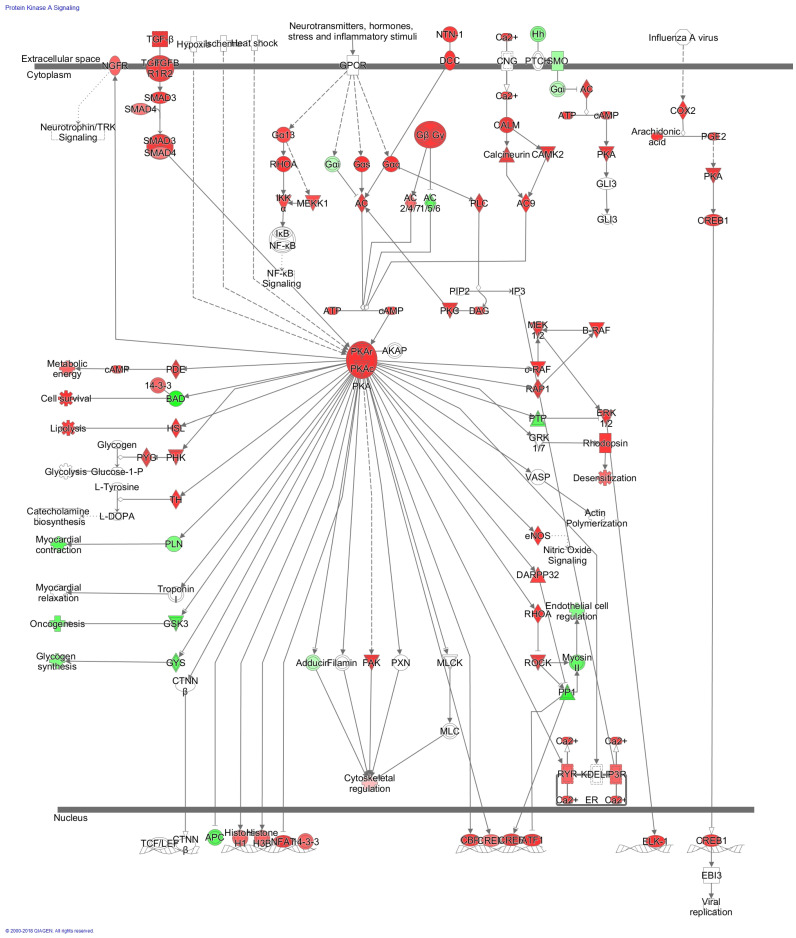
The neurotrasmitters, hormones, stress, and inflammatory stimilus induced activation of protein kinase A (PKA) signaling pathways and multiple effects on metabolic energy, lipolisis, glycogen synthesis, oncogenesis, cell survival, and other canonical adaptive signaling pathways [246].

**Figure A8:**
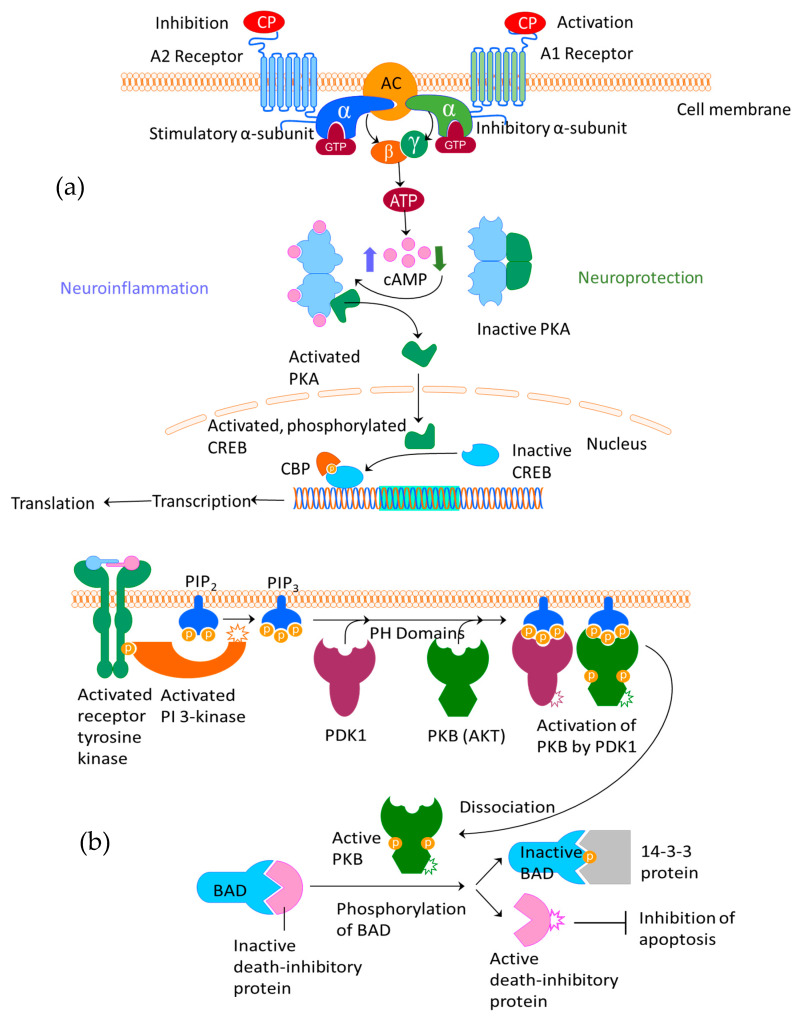
(**a**) Neuroprotective effects of cordycepin on activation of cAMP, PKA and cAMP-response element binding protein (CREB) in neuroinflammation [59,248], and (**b**) PI3-kinase and PKB (AKT) signaling of inhibition of apoptosis that promotes cell survival.

Published pharmacokinetic studies of cordycepin and adenosine show remarkable variability, with rapid metabolism, low oral bioavailability, and plasma concentrations often in the low nanomolar range [127,128,131,132]. These levels are insufficient to activate classical high-affinity adenosine receptors or produce direct pharmacological effects via receptor agonism or antagonism (Section 2.2). Purinergic receptors are important for regulating inflammation, muscle contraction, neurotransmission, and nociception. Extracellular ATP and its metabolites are the main ligands for these receptors. ATP supplementation in healthy subjects for 4 weeks did not change blood or plasma ATP concentrations. Of all ATP metabolites, only plasma uric acid levels increased significantly after the administration of 5000 mg of ATP [85]. A single oral dose of ATP is not bioavailable in healthy human subjects, which may explain why several studies failed to detect ergogenic effects of oral ATP supplementation. On the other hand, increases in uric acid after release of ATP in the proximal part of the small intestine suggest that ATP or one of its metabolites is absorbed and metabolized [84]. This implies that extensive metabolism has no effect on the bioactivity of accumulated ATP in intracellular processes, and particularly on the AMPK-mediated signaling pathway, which plays an essential role in stress response, cellular senescence, apoptosis, autophagy, angiogenesis, protein and glycogen synthesis, steroids biosynthesis, mTOR signaling, etc. [59,248] (Appendix A Figure A6, Figure A7 and Figure A8). Similarly, 3-deoxyadenosine (cordycepin), even at physiological concentrations, may compete with adenosine for receptors, thereby triggering AMPK- and AKT (protein kinase B)-mediated intracellular signaling pathways. Some predicted kinase interactions may not be directly achievable at physiological concentrations, and indirect or systems-level mechanisms may contribute to biological activity. Further studies are required, as physiological concentrations of cordycepin are essential for experimental evidence supporting our hypothesis. For comparison, Ginsenoside Rg5 in physiological concentrations (nM-pM-fM) significantly deregulates gene expressions in brain cell culture [173].

These nucleosides fall within the range where hormetic activation of metabolic stress-response pathways, such as AMPK, SIRT1, TIGAR, and PGC-1α, occurs. Thus, the inconsistencies in pharmacokinetic and pharmacodynamic characteristics of cordycepin are not contradictory but relatively entirely consistent with the concept that cordycepin functions as a metabolic adaptogen with a biphasic hormetic dose–response. Cordycepin may act as a driver, triggering the organism’s adaptive stress response in stress-induced and aging-related diseases.

The lack of correlation between plasma cordycepin concentration and biological outcomes indicates that it does not act through linear, dose-dependent pharmacology but rather through multi-target metabolic adaptation, characteristic of adaptogenic botanicals. In this context, even low, transient, and variable systemic exposures can induce stable improvements in transcription and mitochondrial function.

Cordycepin exhibits a genuine adaptogenic profile driven by low-dose hormetic activation of metabolic resilience pathways, particularly AMPK, SIRT1, PGC-1α, and TIGAR. Although it lacks CNS stimulant effects and does not act via adenosine receptors, cordycepin enhances mitochondrial efficiency, improves ATP production, and increases resistance to physical and metabolic stress. Within *C. militaris* or *O. sinensis* extracts, synergistic interactions among multiple constituents amplify these effects, providing a biologically plausible explanation for the anti-fatigue and performance-enhancing outcomes observed in animal and human studies.

Cordycepin exerts effects through peripheral metabolic enhancement, mitochondrial biogenesis, and hormetic stress adaptation rather than through central adenosinergic stimulation. Cordycepin acts as a low-dose metabolic hormetic agent, while Orange Cordyceps and Caterpillar Fungus extracts display multi-target synergy that cannot be explained by nucleosides alone. This framework resolves the apparent contradictions between cordycepin’s limited CNS pharmacology and the robust anti-fatigue outcomes observed in animals and humans.

### 3.3. Western Regulatory vs. Traditional Medical Paradigms

TCM and Ayurveda are based on a holistic and integrative approach, multi-target and polyvalent action [134], but they rely on archaic theories. In contrast, Western conventional medicine, based on a reductional approach, selective targeting, and specific action in the treatment of diseases, relies on another archaic theory of galenic preparations that ignores interactions and overlaps among regulatory systems and the effects of other constituents in multi-component plant extracts. In fact, both approaches are complementary, particularly in the treatment of diseases of complex pathology associated with the stress system interacting with other regulatory systems. The concomitant administration of several Western conventional medicines that selectively act on various receptors provides more effective treatment in hypertension, post-stroke rehabilitation, viral infections, etc. The “ready for use” of complex botanicals and botanical hybrid products used in the Eastern world acts similarly. The challenge is to understand the mechanisms of action and scientifically validate their efficacy, quality, and safety by strengthening the integration of TCM into European health systems, improving quality control, conducting randomized clinical trials, and improving regulatory standards [249].

The concept of adaptogens originated in Eastern medical and pharmacological traditions and describes natural products that increase an organism’s capacity to maintain physiological homeostasis under conditions of stress [28,135]. In this framework, stress is understood as a multisystem challenge that affects neuroendocrine regulation, energy metabolism, immune competence, and cognitive and physical performance, particularly in aging and stress-exposed populations [229,230,250,251,252,253,254,255,256].

While adaptogens are commonly recognized as a pharmacological category in several non-European regulatory and medical systems [256,257,258,259,260,261,262,263,264], they are not formally acknowledged within EFSA or EMA frameworks. The term “adaptogen” is considered not appropriate for a marketing authorization within EFSA or EMA frameworks [241], which require substantiation of discrete, well-defined physiological endpoints and do not evaluate systems-level concepts such as adaptation or stress resilience [238,239,240]. As a consequence, holistic or systems-level constructs such as “adaptation”, “stress resilience”, or “anti-fatigue” cannot be evaluated as such under current EU food or medicinal product legislation.

Importantly, the lack of regulatory recognition does not imply scientific invalidity of the adaptogen concept but rather reflects methodological constraints of existing regulatory paradigms.

In summary, the adaptogen concept represents a systems-level interpretation of complex physiological responses, whereas EFSA evaluation operates at the level of single, well-defined functional outcomes. By translating adaptive effects into EFSA-recognized physiological functions—such as physical performance or perceived exertion—it is possible to preserve scientific validity while remaining compliant with regulatory requirements.

This translational framework enables constructive dialogue between scientific innovation and regulatory practice, without forcing premature or inappropriate reclassification of complex biological concepts.

### 3.4. Resilience Biology as the Missing Regulatory Construct

The findings of this review indicate that the regulatory marginalization of adaptogens in Europe does not arise from a lack of biological plausibility, but from a structural gap in regulatory concepts. Inflammation and stress responses are both evolutionarily conserved adaptive systems with active resolution mechanisms [229,230,231,232,233,234,235,236]; however, only inflammation is formally recognized as a regulatable pathological entity within European frameworks [238].

Adaptogenic botanicals consistently demonstrate modulatory effects across immune, inflammatory, neuroendocrine, and metabolic systems [26,28,134]. These effects are bidirectional, context-dependent, and primarily aimed at restoring homeostasis rather than suppressing specific pathways. Such characteristics align poorly with disease-centric regulatory logic but are fully consistent with contemporary systems biology, allostasis, and resilience theory [232,265].

Asian medical systems have long operationalized this concept by framing health as adaptive capacity rather than the absence of disease [244]. In contrast, European regulation—despite recognizing allostasis, inflammation resolution, and psychoneuroimmunology at the scientific level—lacks a functional category that allows these principles to be translated into health claims or product classifications [238,239,240]

#### 3.4.1. Why a Hybrid EU–Asia Model Is Scientifically Justified

A hybrid EU–Asia regulatory model reconciles two complementary strengths: the European emphasis on safety, standardization, and evidentiary rigor, and the Asian emphasis on functional balance, recovery, and resilience embedded in traditional medical systems [26,28,134,244,264,265].

This review demonstrates that adaptogens act upstream of pathology by supporting recovery kinetics, physiological flexibility, and stress-response resolution—features increasingly recognized as determinants of long-term health outcomes [232,265]. These effects are not adequately captured by current EU categories, such as vitamins, antioxidants, or anti-inflammatory agents, which are anchored in deficiency-correction or pathway-inhibition models [238,239,240]. Nevertheless, adaptogenic effects can be measured using non-disease endpoints, including return-to-baseline time, performance maintenance under load, and immune homeostasis markers [26,28].

Importantly, the proposed hybrid model does not dilute regulatory rigor. Rather, it reallocates evidentiary focus from disease endpoints to functional adaptation metrics, maintaining consumer protection while enabling scientific innovation consistent with systems biology.

##### 3.4.1.1. Resilience-Supporting Physiological Modulators as a Regulatory Bridge

The proposed category of resilience-supporting physiological modulators (RSPMs) offers a legally conservative yet biologically modern solution. By explicitly excluding disease prevention or treatment claims, this category preserves the boundary between food supplements and medicines while legitimizing claims related to recovery, homeostasis, and adaptive capacity. Such a category would be compatible with the existing institutional roles of the European Food Safety Authority—responsible for safety evaluation and health-claim substantiation—and the European Medicines Agency, which oversees the boundary between functional and pharmacological activity [238,239,240,241]. Asian regulatory frameworks would serve as functional reference models rather than direct validation pathways, allowing integration without undermining EU legal coherence.

Further expert consensus guidelines can be developed to focus on the selection and justification of outcome measures and endpoints across various stress-induced and aging-related health conditions, and to propose measurable resilience-related biomarkers as candidate endpoints to operationalize the proposed resilience-supporting physiological modulator (RSPM) framework. The criteria for resilience and the biomarker set are similar to those summarized in a recent review on adaptogens [26], including recovery indices in chronic unpredictable stress (CUS) animal models, which induce affective behaviors in mice and, once established, measure stress-related alterations in the intrinsic excitability and synaptic regulation of the medial prefrontal cortex layer pyramidal neurons, cortisol recovery kinetics, cytokine balance ratios, and adaptive stress–response signaling pathways markers, e.g., G-protein coupled (GPCR), tyrosine, toll-like receptors, and I3PK-AKT, AMPK-mediated pathways known to promote survival in response to stress, suggesting neuroprotective activity and the potential benefits of adaptogens in neurodegenerative diseases.

##### 3.4.1.2. Regulatory Language as a Determinant of Scientific Visibility

One of the essential points of this review is that language—not evidence—is the principal barrier to adaptogen acceptance for marketing authorization in Europe. Terms such as anti-stress or adaptogenic for marketing authorization are rejected not because stress biology is unrecognized, but because stress is classified as a normal life condition rather than a regulatable physiological domain [238,239,240,241]. The hybrid model resolves this tension by shifting the claim architecture toward supporting normal physiological function during periods of increased demand, a formulation already consistent with European health-claim jurisprudence. This reframing allows resilience biology to be communicated without encroaching on medical or psychological claims, thereby increasing regulatory acceptability while preserving scientific meaning.

#### 3.4.2. Outlook and Future Directions

##### 3.4.2.1. From Disease Regulation to Health Maintenance

European regulatory systems are approaching a conceptual inflection point. As chronic, lifestyle-associated conditions increasingly dominate healthcare burden, the inability to regulate interventions that support recovery and adaptive capacity represents a growing limitation (Furman et al., 2019) [265]. Resilience biology provides a scientifically grounded framework to address this gap.

The hybrid EU–Asia model outlined here offers a pathway to transition from a purely disease-reactive paradigm toward health-maintenance regulation, without compromising evidentiary standards or public safety.

##### 3.4.2.2. Implications for Regulatory Science

Future regulatory development should prioritize:

- Formal recognition of homeostasis and recovery as legitimate physiological outcomes;
- Acceptance of systems-level functional endpoints alongside classical biomarkers;
- Development of claim guidance specific to resilience-supporting functions.

Pilot implementation through emerging-science claim pathways, limited botanical lists, and post-market monitoring would allow regulatory learning while maintaining risk control [238,239,240].

##### 3.4.2.3. Implications for Research and Industry

Adoption of a resilience-focused regulatory category would incentivize improved human study designs using load-and-recovery paradigms, better standardization of complex botanical preparations, and closer integration of traditional knowledge with modern clinical research [26,244]. This would reduce the current disconnect between scientific evidence, regulatory acceptance, and consumer communication.

##### 3.4.2.4. Concluding Perspective

The regulatory challenge posed by adaptogens is not an anomaly but a signal: modern biology has outgrown purely disease-centric regulation. A hybrid EU–Asia regulatory model integrating resilience biology would align policy with science, recognize health as a dynamic adaptive process, and provide a coherent framework for interventions that maintain function rather than treat pathology.

Resilience is not an alternative to medicine; it is the biological foundation upon which medicine succeeds.

## 4. Materials and Methods

This mixed-methods review explores evidence from 9 systematic clinical and 37 network pharmacology studies on *O. sinensis*, *C. militaris*, and cordycepin, extracting active compounds, disease indications, predicted targets, enriched signaling pathways, and mechanistic conclusions to elucidate their molecular actions in diseases treated in TCM. A systematic review of meta-analyses of randomized controlled trials (RCTs) was conducted to evaluate the efficacy, safety, quality control practices, and compliance with EMA criteria for well-established herbal medicinal products. Data synthesis included pooled effect-size estimation and risk-of-bias assessment using Cochrane methods. A structured umbrella review approach was applied to peer-reviewed reviews addressing inflammation, stress physiology, adaptogens, and regulatory science. Regulatory guidance from EFSA, EMA, FDA, and Asian authorities was examined, and findings were narratively synthesized.

### 4.1. Literature Search

This review thoroughly explores the current evidence on the network pharmacology studies of *O. synensis*, *C. militaris*, and cordycepin, covering publications from 2010 to 2025 and addressing inflammation, stress physiology, adaptogens, and regulatory science. Regulatory guidance from EFSA, EMA, FDA, and Asian authorities was examined. Findings were narratively synthesized. A search was conducted using several major databases and platforms, including PubMed, Google Scholar, ScienceDirect, Scopus, CNKI, and Web of Science, as well as the Google search engine, the National Library of Medicine, regulatory documents from EFSA, EMA, FDA, and Asian health authorities, and the ChatGPT AI technology.

The search strategy involved targeted use of keywords such as “*Ophiocordyceps sinensis*”, “*Cordyceps militaris*” (L.), “network pharmacology,” “randomized” AND “health claims”, “indications” AND “pharmacopeia”, AND “dietary supplements”. The search was limited to English-language publications within the specified period. The review process adhered to EMA guidelines, and the quality of the studies was evaluated using CONSORT and Cochrane standards.

### 4.2. Inclusion and Exclusion Criteria

#### 4.2.1. Inclusion Criteria

For network pharmacology synthesis:

- Studies explicitly performing target prediction and pathway enrichment analyses.
- Studies identifying protein–protein interaction (PPI) networks.
- Studies reporting enriched KEGG/GO pathways.
- Studies involving *O. sinensis*, *C. militaris*, or cordycepin as primary exposure.

For experimental validation:

- In vitro or in vivo confirmation of predicted targets/pathways.
- Clear reporting of biological endpoints.
- Mechanistic linkage to predicted signaling hubs.

For clinical evaluation:

- Randomized controlled trials (RCTs).
- Systematic reviews or meta-analyses.
- Clearly defined preparation type.

#### 4.2.2. Exclusion Criteria

- Reviews without primary mechanistic data.
- Studies lacking explicit pathway enrichment methods.
- Case reports without mechanistic evaluation.

### 4.3. Data Extraction

Data extraction covered: major active compounds/ligands, disease indications, predicted molecular targets, enriched signaling pathways, docking scores, and presence of experimental validation. Target convergence and pathway frequency analyses were performed to identify core mechanistic hubs.

Included studies employed network pharmacology tools such as target prediction algorithms, KEGG/GO pathway enrichment, molecular docking, transcriptomics, metabolomics, or integrated multi-omics. Ten studies with experimental validation—including cellular assays, animal disease models, and clinical observations—were also included.

Reviews addressing inflammation resolution, stress biology, adaptogens, or regulatory frameworks were also included. Findings were narratively integrated across biological, clinical, and regulatory domains.

### 4.4. Data Integration and Evidence Integration Strategy

All AI-assisted frequency aggregation was manually verified against original publications to prevent duplication or misclassification.

Thirty-seven network pharmacology studies were identified. For each study:

- Predicted targets were extracted.
- Enriched pathways were recorded.
- Frequency of pathway recurrence across independent studies was counted.

To reduce “target inflation bias,” predicted targets were stratified according to validation level:

- In silico prediction only;
- Experimentally validated (cellular or animal models);
- Clinically supported endpoints.

Targets and pathways were aggregated across studies. Mechanistic convergence was assessed by frequency. Experimental studies were cross-mapped onto predicted networks. Heatmaps (Figure 4) represent recurrence frequency across independent publications, not gene expression magnitude.

AI-assisted tools were used exclusively for structured aggregation and visualization of manually extracted data. Literature identification, study selection, and data extraction were performed manually from full-text publications. Extracted variables (intervention, targets, enriched pathways, validation status, and study identifiers) were compiled into standardized tables (Table A10 and Table A11) prior to AI processing. AI was subsequently used only to compute recurrence frequencies, stratify findings by evidence tier, and generate graphical heatmaps (Figure 4). No automated literature screening, target prediction, or mechanistic interpretation was performed by AI. All aggregated outputs were manually cross-verified against the original dataset to prevent duplication or misclassification.

## 5. Conclusions

Many reviews on various aspects of the medical mushroom *O. sinensis*, which has historically been recognized in Asian traditional medical systems for its ability to enhance vitality, describe pleiotropic therapeutic applications. However, the polyvalent adaptogenic potential of *O. sinensis* has not been systematically proven, and a consolidated mechanistic synthesis integrating both network predictions and experimental validation is lacking. This review, for the first time, shows that:

- *O. sinensis*, *C. militaris*, and cordycepin share a common adaptogenic mechanism of maintenance of cellular and integrated biology system functions homeostasis.
- The systems-level adaptogenic mechanism of these fungi is characterized by their ability to modulate multiple interconnected biological networks rather than acting on a single target.
- This is in line with TCM and Ayurveda holistic concepts and the modern concept of pleiotropic therapeutic activity of adaptogens and particularly of *O. sinensis*.
- The review reveals controversy regarding the bioavailability of cordycepin in vivo and its concentration in vitro studies, raising the hypothesis that cordycepin may act as a driver, triggering the organism’s adaptive stress response in stress-induced and aging-related diseases.
- Nucleosides, adenosine and cordycepin, along with other adaptogenic botanical metabolites (steroids and phenolics), contribute to the maintenance of cellular and integrated biology system functions homeostasis.
- Network pharmacology studies identify multi-target pathways, including convergent hubs, such as PI3K-Akt, AMPK–mTOR, MAPK, apoptosis, Nrf2 and AMPK–SIRT1–PGC-1α pathways.
- By influencing pathways associated with immune regulation, mitochondrial function, and metabolic adaptation, they orchestrate a holistic response that enhances the organism’s resilience to various stressors.
- This integrative effect is especially significant in the context of complex disorders, where multifactorial interventions are necessary for effective therapeutic outcomes.
- This is the first comparative meta-analysis of validated vs. predicted effects of *O. sinensis*, *C. militaris* and species-level versus cordycepin-driven mechanisms.
- Validation studies confirm predictions across chronic obstructive pulmonary disease, pulmonary arterial hypertension, cancer, obesity, influenza, and immunogenicity.

Caterpillar Fungus, Orange Cordyceps, and 3-deoxyadenosine exhibit a unified adaptogenic pharmacology that involves immune modulation, mitochondrial enhancement, stress-signal regulation, and metabolic homeostasis. These effects arise through multi-target synergy across PI3K–Akt, MAPK, NF-κB, apoptosis, and AMPK–SIRT1–PGC-1α pathways. The meta-analysis of network pharmacology studies suggests that *O. sinensis* and *C. militaris* act as multi-compound immunometabolic regulators, while cordycepin functions as a dominant molecular effector controlling apoptosis, metabolism, and adaptive stress signaling. The strong convergence of network and experimental evidence supports their potential in immunometabolic and stress-related disorders. Distinguishing validated from predicted networks is essential for translational reliability.

Results of network pharmacological studies of OS, CM, and CC that demonstrate activation of adaptive extracellular and intracellular signaling pathways, key mediators, and physiological functions are associated with pleiotropic health effects.

Clinical efficacy of OS, CM, and CC is noticed in stress-induced physical and cognitive fatigue, mental and immune disorders; these studies are limited to physical fatigue in healthy individuals, chronic kidney disease, respiratory conditions, and cancer adjunct therapy.

The effects of cordycepin were demonstrated in micromolar concentrations in vitro cell models. Clinical and pharmacokinetic studies show that high concentrations of cordycepin, adenosine, and their bioactive metabolites, ATP and CTP, in blood circulation cannot be achieved with therapeutic doses of *O. sinensis* and *C. militaris* due to intensive enzymatic metabolism and renal clearance. However, since ATP, adenosine, cordycepin, and other purine metabolites are involved in numerous physiological processes and exert pharmacological effects, we can hypothesize that they trigger these effects at physiological nanomolar concentrations. Cordycepin may function as a systems-level metabolic trigger rather than as a classical high-affinity receptor ligand.

The adaptogenic concept represents a systems-level interpretation of complex physiological responses characteristic of holistic TCM and Ayurvedic concepts, whereas European Food Safety Authority regulatory frameworks lack concepts for resilience and adaptive capacity and operate at the level of single, well-defined functional outcomes, which raises regulatory issues for food and drugs in Western countries.

This review clarifies conceptual and regulatory barriers to recognizing resilience-supporting interventions and informs future regulatory innovation. The review for the first time suggests establishing a regulatory category for resilience-supporting physiological modulators that could align food and drug regulation in the EU with contemporary systems biology, thereby complementing EFSA, EMA, FDA, and Asian authorities.

Numerous critical appraisals and limitations were discussed above in detail across the sections on clinical and network pharmacology, as well as in the regulatory framework. Future perspectives of primary importance include integrating TCM into European health systems, improving quality control, conducting randomized clinical trials effectively, strengthening regulatory standards, and the wide implementation of network pharmacology study design and methodology, which account for synergistic and antagonistic interactions by various constituents of the complex, multi-component extracts, which can lead to unexpected outcomes. They include transcriptome-wide microarray profiling of gene-expression-based experiments, integrated with metabolomics and network analyses, revealing all molecular targets of active compounds and coupling signaling pathways to final outcomes. The results of these studies can reveal unknown health consequences and therapeutic indications, leading to new drug discoveries.

## Figures and Tables

**Table 1:** Key analytical markers and typical ranges in *O. sinensis* and *C. militaris* products.

Markers	*O. sinensis*, mg/g	*C. militaris*, mg/g	References
Adenosine	≈0.2–10	0.3–2.5	[86,87,88]
Cordycepin	<0.2 (0.006–0.075)	~6.6 (3–26)	[53,87,88,89,90]
Polysaccharides	≥60 (≈50–100)	≥80 (~30–150)	[16,88,91]
Total nucleosides	~2.0–3.1		[92,93,94]
Total Ergosterol	~1.9–2.7	~2–3	[95,96]
Mattitol	25.6–115.7		[89,97]

**Table 2:** Mental and physical fatigue effects.

Compound	Receptor Action	Net CNS Effect	Fatigue Impact	Mechanistic Reason
Adenosine	Agonist at A1, A2A	Sedative	Increases fatigue	Inhibits neuronal firing, promotes sleep pressure
Caffeine	Antagonist at A1, A2A	Stimulant	Reduces fatigue	Blocks adenosine, increases dopamine + cortical activity
Cordycepin	Minimal activity (in vivo)	Neutral	No proven effect	Rapid deamination, poor BBB penetration, weak receptor affinity

**Table 3:** Summary of clinical evidence from systematic reviews of *O. sinensis* and grading against EMA well-established use criteria.

References	Main Preparations Studied	Indications (Systematic Reviews)	No. of SRs/Meta-analyses	Approx. No. of RCTs (Patients)	Consistency of Clinical Effects	Methodological Quality Certainty of Evidence	Key Limitations	EMA WEU Relevance
Liu et al., 2024; Wu et al., 2025 Pu et al., 2024; Tao et al., 2024; Xue et al., 2024; [136,137,138,139,140]	Fermented mycelium (Bailing, Jinshuibao, Zhiling, others)	Dialysis (HD/PD), DKD, CKD, CA-AKI prevention, renal dysfunction	5	>210 RCTs (>16,000 pts, overlapping);	Consistent improvement in renal and inflammatory surrogate markers (Scr, BUN, CRP, proteinuria); reduced CA-AKI incidence when used preventively	Low to very low (GRADE); majority of SRs critically low (AMSTAR-2)	Open-label RCTs; short follow-up; surrogate endpoints; heterogeneous preparations	Does not meet WEU (efficacy signal present, but insufficient robustness and preparation definition)
Wang et al., 2024; Yu et al., 2019; Ma & Jin, 2024 [141,142,143]	Bailing capsule, cultivated mycelia, mixed CS preparations	COPD, lung cancer (adjunctive),	3	~80–100 RCTs (>5000 pts)	Consistent adjunctive benefits (lung function, QoL, immune markers, tumor response rate)	Low to moderate	Lack of blinding; all trials conducted in China; unclear allocation concealment; adjunctive designs only	Does not meet WEU (methodological and EU-use limitations)
Welch et al., 2023; Dewi & Khemtong, 2025 * [144,145]	Supplements (varied)	Healthy volunteers/exercise performance	2	7 RCTs (286 pts)	Inconsistent, small effects	Low	Small samples; heterogeneous outcomes; limited clinical relevance	Not relevant for WEU

Abbreviations: CA-AKI, contrast-associated acute kidney injury; CKD, chronic kidney disease; DKD—diabetic kidney disease; CRP, C-reactive protein; HD, haemodialysis; PD, peritoneal dialysis; QoL, quality of life; RCT, randomized controlled trial; Scr, serum creatinine; SR, systematic review; WEU, well-established use. *—Lack of preferred reporting items for systematic reviews and meta-analyses statement (PRISMA).

**Table 4:** Characteristic differences in in silico prediction-only (Tier 1) * and experimentally validated (Tier 2) network pharmacology studies and clinical alignment (Tier 3) of *O. sinensis*, *C. militaris* preparations, and cordycepin.

Feature	*O. sinensis*	*C. militaris*	Cordycepin
Main bioactive constituents	Adenosine/2′-deoxyadenosine, ergosterol, adenosine, glutamine, diverse peptides	Cordycepin (3′-deoxyadenosine), adenosine, polysaccharides, ergosterol	Cordycepin
Dominant validated indications (Tier 2)	COPD, lung cancer, CKD, ischemic stroke, diabetic nephropathy, UV injury, vaccine adjuvant (Tier 3)	Immune modulation, lung cancer, COPD, gouty nephropathy, SARS-CoV-2	Cancer, obesity, depression, PAH, immunosuppression, glioblastoma
Main predicted indications (Tier 1)	Liver cancer, DN, hypoxia, depression, PCOS, influenza	COVID-19 inflammation	Cancer, Alzheimer’s, kinase inhibition
Signature pathways (Tier 2)	PI3K-Akt, TNF, AGE-RAGE, IL-17, chemokine, apoptosis, lipid metabolism	TLR, inflammatory lipid pathways, apoptosis, oxidative stress, metabolic regulation	Apoptosis, AMPK-mTOR, PI3K-Akt, Nrf2, Wnt/β-catenin, metabolic reprogramming
Predicted pathways (Tier 1)	AGE-RAGE, VEGF, PI3K-Akt, FOXO, neurotrophic, inflammatory	A3AR, JNK, AMPK, AKT, MAPKs, FOXO3	Apoptosis, neurodegeneration, and kinase inhibition
Core hubs repeatedly validated (Tier 2)	AKT1, CASP3, PTGS2, HIF1A, NFKB1, VEGFA, PTEN, STING, CXCR4,	TLR4, TNF, EGFR, IDH1, PTGS2, ABCG2, p53	AKT1, CASP3, EGFR, Nrf2, GSK3β, β-catenin, FOXO, MAPKs
Core predicted hubs (Tier 1)	TNF, MAPK1, EGFR, IL6, VEGFA, ACE, CASP3	NR3C1, thrombin, inflammatory kinases	A3AR, JNK, AMPK, AKT, MAPKs, FOXO3
Key validated differences (Tier 2)	immune-pulmonary-renal axis, lipid/steroid mediation, inflammatory microenvironment remodeling (Tier 3)	immune activation, metabolic enzymes, antiviral/anticancer lipid-nucleoside networks	intracellular stress signaling, kinase control, apoptosis, metabolic rewiring

(*)—Tier 1 studies expand hypothesis space but lack biological confirmation, Appendix A Table A11.

**Table 5:** Adaptive stress–response signaling pathways and physiological role modulated by cordycepin.

Pathway/Module	Key Genes/Molecules	Physiological Roles	Representative Publications
AMPK–mTOR–Autophagy Axis	PRKAA1/2 (AMPKα), RPTOR, RICTOR, MTOR, ULK1, BECN1, LC3B, ATG5/7, SQSTM1	Cellular energy sensor; promotes autophagy, ATP conservation, and repair during stress.	Hawley et al., 2020 [36] Marcelo et al., 2019 [209] Li et al., 2017 [210].
NRF2–KEAP1 Antioxidant Axis	NFE2L2 (NRF2), KEAP1, HMOX1, NQO1, GCLC, GCLM, SOD2, CAT, GPX1	Regulates antioxidant enzymes, detoxification, redox homeostasis, and cytoprotection.	Wang Z et al., 2019 [211]
TLR4–MyD88–NF-κB/MAPK Inflammatory Axis	TLR4, MYD88, NFKB1, RELA, IKBKB, MAPK14, MAPK8, MAPK1/3, TNF, IL6, PTGS2, NOS2	Controls innate immune activation; cordycepin suppresses pro-inflammatory cytokines and iNOS/COX-2 expression.	Choi YH et al., 2014 [212] Yang J et al., 2017 [213] Sun Y et al., 2020 [214]
NLRP3 Inflammasome/Pyroptosis Regulation	NLRP3, CASP1, GSDMD, IL1B, IL18	Limits pyroptotic cell death and IL-1β release; anti-inflammatory and neuroprotective effects.	Liu Z et al., 2025 [215] Zhang X et al., 2021 [216]
PI3K–AKT/FOXO–SIRT Pathway	PIK3CA/B, AKT1, FOXO3, SIRT1, TP53	Balances survival vs. repair; mediates stress resistance, longevity, and metabolic adaptation.	Li T et al., 2019 [217]

**Table 6:** Key genes involved in cordycepin-induced adaptive stress–response signaling pathways and their physiological roles.

Signaling Pathway	Key Genes Involved	Physiological Role
AMPK pathway	AMPK, SIRT1, MTOR	Energy sensing, lifespan extension
Sirtuin signaling	SIRT1, FOXO3, PARP1	Regulates stress response, aging,
mTOR signaling	MTOR, AMPK, SIRT1, TP53	Nutrient sensing, autophagy, and protein synthesis
DNA damage response	TP53, PARP1	Genomic stability, cancer prevention

**Table 7:** Regulatory classification of “Cordyceps” mushroom in the EU, USA, China, Japan, and the Republic of Korea.

Region	*O. sinensis* *	*C. militaris*	Notes
China	Traditionally medicinal, wild OS is not a general everyday food. Listed in the Chinese *Pharmacopeia* as a traditional medicinal material (TCM drug), not a general food. *Ophiocordyceps sinensis* mycelium and fermented products (some strains) may appear as approved food ingredients if separately registered.	Approved as a “new resource food” (novel food ingredient) since 2009; widely used in foods/health foods in China.	Used in decoctions, capsules, and medicinal soups. Only specific strains of cultivated mycelium (e.g., Cs-HK1) are allowed as food supplements. Contemporary reviews of the Chinese regulatory framework note *C. militaris* approval as novel/new resource food (2009) [218].
Japan/Republic of Korea	Classified as a traditional medicinal material, used in Kampo or functional foods under regulatory oversight.	In Asian countries, the cultural and historical use supports a more permissive edible status for *C. militaris*	In Asian countries, the cultural and historical use supports a more permissive edible status for *C. militaris*
USA	Marketed mainly as dietary supplements (mycelium/extracts). No GRAS (Generally Recognized As Safe) listing for wild *O. sinensis*; marketed forms are dietary supplements under DSHEA, typically using cultivated mycelium (e.g., *O. sinensis* Cs-4).	Marketed as dietary supplements, enforcement actions are taken when drug-like claims are made.	The U.S. classifies many mushroom extracts as dietary supplements. If a product claims to treat a disease, the FDA may treat it as a drug. U.S. Food and Drug Administration. FDA warning letters show Cordyceps products can be challenged if marketed with disease claims. There is no public GRAS for wild OS itself.
EU	Considered a novel food if intended as a food ingredient, wild *O. sinensis* itself is not authorized under general edible mushroom lists. Not novel in food supplements (entry in the EU Novel Food status catalog).	“Not yet authorized–novel food.”	Authorization required under the Novel Foods Regulation. The European Commission’s Novel Food portal lists *C. militaris* (mycelium and fruiting body) as not yet authorized; multiple RASFF alerts flag unauthorized use of *C. militaris* in supplements. *O. sinensis* is shown as “not novel in food supplements.”
Sweden	Follows EU Novel Food rules.	Follows EU Novel Food rules.	In the EU, Novel Food law is harmonized; Sweden applies the EU stance. Check the EC catalog and consult the Swedish National Food Agency if you need product-specific confirmation.

* Safety considerations: (i) Natural wild *O. synensis* may accumulate arsenic and heavy metals from high-altitude soils; limits have been noted in Chinese safety advisories, (ii) cultured mycelium (fermentation products) avoids this risk and is the basis of most approved “Cordyceps” food supplements (e.g., Cs-4 strain), (iii) no known severe allergic risks at standard doses, but data remain limited.

**Table 8:** Similarities and differences between inflammation and stress responses.

Aspect	Inflammation	Stress Response
Purpose	Restore homeostasis	Restore homeostasis
Nature	Protective, adaptive	Protective, adaptive
Mediators	Cytokines, eicosanoids	Hormones, neurotransmitters
Resolution	Actively regulated	Actively regulated
Pathology arises when	Chronic, unresolved	Chronic, dysregulated
Localization	Primarily local	Systemic
Primary system	Immune system	Neuroendocrine system
Measurability	Clear biomarkers (CRP, IL-6)	Context-dependent biomarkers (cortisol variability)
Disease framing	Recognized pathological entity	Often framed as a risk factor
Consequences of failed resolution	Chronic inflammation: Cardiovascular disease Autoimmune disorders Neurodegeneration Cancer progression	Chronic stress/inflammaging: Depression and anxiety Metabolic syndrome Immune suppression or hyperactivation Accelerated aging

**Table 9:** Regulatory acceptance of adaptogens: EU vs. US vs. Asia.

Dimension	European Union	United States	Asia (China, Japan, the Republic of Korea, India)
Adaptogen term	Not recognized	Informally tolerated	Explicitly recognized
Regulatory model	Disease- & nutrient-centric	Risk-based consumer access	Systems & functional medicine
Acceptable claim style	Narrow, mechanistic	Structure–function	Functional, pattern-based
Stress-related claims	Rejected	Allowed with disclaimer	Allowed
Traditional use	Secondary	Optional	Central

**Table 10:** Western regulatory vs. traditional medical paradigms: core worldview difference.

Dimension	Western Regulatory Paradigm (EFSA/EMA)	Traditional Medical Paradigms (TCM, Ayurveda, etc.)
Primary goal	Treat or prevent a defined disease	Restore balance and resilience
Model	Reductionist, mechanistic	Systems-based, integrative
Body concept	Sum of organs and pathways	Dynamic, interconnected whole
Health	Absence of pathology	Capacity to adapt and recover
Evidence	Isolated endpoints, biomarkers	Pattern recognition, outcomes over time
Disease vs. dysfunction	Requires: A defined pathological condition Clear causal pathway Measurable, reproducible endpoints Works well for: Inflammation Deficiency diseases Infections	Focuses on: Functional imbalance before disease Fatigue, low vitality, stress intolerance Recovery capacity
Evidence hierarchy mismatch	Standardized extracts Dose–response relationships Single or narrow endpoints	Multi-target effects Long-term use Context-dependent outcomes Synergistic formulations

## Data Availability

No new data were created or analyzed in this study.

## Appendix A

### Appendix A.1. Taxonomy, Scientific and Vernacular Names of Ophiocordyceps sinensis and Cordyceps militaris

*Ophiocordyceps sinensis* is a species of ascomycete fungi in the family Ophiocordycipitaceae.

*Cordyceps militaris* is a species of ascomycete fungi in the family Cordycipitaceae.

**Table A1:** Scientific and vernacular names of *Ophiocordyceps sinensis* and *Cordyceps militaris*.

Accepted Scientific Name	*Ophiocordyceps sinensis* (Berk.) G.H. Sung, J.M. Sung, Hywel-Jones & Spatafora, 2007	*Cordyceps militaris* (L.) Fr., 1818
*Synonims*	*Cordyceps sinensis* (Berk.) Sacc. (1878), *Sphaeria sinensis* Berk. (1843)	*Clavaria militaris* L., 1753
Language	Vernacular names	
Sanskrit	Yarsagumba	
Tibetan	Yarsa gumba, Yarcha gumba (དབྱར་རྩ་དུད་འབྱུར་)	
Nepali	Keera jhar, Jeevan buti, Keeda ghass, Chyou kira, Sanjeevani bhooti (यार्सागुम्बा)	
Chinese (Mandarin)	Dong chong xi cao (冬蟲夏草, or 冬虫夏草, meaning “winter worm, summer grass”)	Běi chóng cǎo (北虫草) Northern Cordyceps
Japanase	Tocheikasa, Tochyuka-sou	冬虫夏草 (Tōchūkasō)
Korean	동충하초 (Dong Chung Ha Cho)	북충초 (Buk chung cho)
Vietnamese	Đông trùng hạ thảo	Đông trùng hạ thảo quân đội
Thai	ถั่งเช่า (Thungchao)	ถั่งเช่าเหนือ (Thungchao nuea)
French	cordyceps, champignon chenille	
German	Cordyceps, Chinesischer Raupenpilz, Tibetischer, Raupenpilz, Tibetischer Raupenkeulenpilz	
Italian	cordyceps, fungo del bruco	
Spanish	cordyceps	
English	Caterpillar fungus, Cordyceps mushroom, Winter Worm—Summer Grass	Scarlet Caterpillar Club, Orange Cordyceps

Etymology: Ophiocordyceps = from Greek ophio- (“snake-like”) + cordyle (“club”) + ceps (“head”). Refers to the slender, serpentine fruiting body; sinensis = Latin for “from China,” where the species was first described (Berkeley, 1843). The traditional phrase “冬虫夏草” (Dōng chóng xià cǎo) encapsulates its dual nature as both animal (insect larva) and plant-like (fungal fruiting body). Cordyceps = from Greek kordyle (club) + ceps (head), militaris = Latin for “military”—a reference to its upright, orange, “soldier-like” appearance.

**Table A2:** Taxonomy of *Ophiocordyceps sinensis* and *Cordyceps militaris*.

Scientific Name	*Ophiocordyceps sinensis* (Berk.) G.H. Sung, J.M. Sung, Hywel-Jones & Spatafora, 2007	*Cordyceps militaris* (L.) Fr., 1818
Domain	Eukaryota (eukaryotes)	Eukaryota (eukaryotes)
Kingdom	Fungi	Fungi
Phylum	Ascomycota (ascomycete fungi)	Ascomycota (ascomycete fungi)
Class	Sordariomycetes	Sordariomycetes
Order	Hypocreales	Hypocreales
Family	Ophiocordycipitaceae	Cordycipitaceae
Genus	*Ophiocordyceps*	*Cordyceps*
Species	*Ophiocordyceps sinensis*	*Cordyceps militaris*

### Appendix A.2. Chemical Composition, Bioactive Compounds, and Pharmacological Activity of Ophiocordyceps sinensis and Cordyceps militaris Species

Overall, 110 primary and secondary metabolites of *O. sinensis* and *C. militaris* fungi were identified in several comprehensive reviews of their traditional uses, chemical composition, and pharmacological activity [16,18,19,69,70,71]. Figure A1, Figure A2, Figure A3, Figure A4 and Figure A5 and Table A3, Table A4, Table A5 and Table A6.

**Table A3:** Chemical bioactive constituents of *O. sinensis* [21,29,65] and *C. militaris* [23,66,67].

*C. sinensis*	*C. militaris*
Cordycepic acid, glutamic acid, amino acids (phenylalnine, praline, histidine, valine, oxyvaline, arginine): polyamines (1,3-diamino propane, cadaverine, spermidine, spermine, homospermidine, and purtescine): cyclic dipeptides (cyclo-(gly-pro), cyclo-(leu-pro), cyclo-(val-pro), cyclo-(ala-leu), cyclo-(alaval), and cyclo-(thr-leu), saccharides and sugar derivatives (d-mannitol, oligosaccharides, and polysaccharides); sterols (ergosterol, delta-3 ergosterol, ergosterol peroxide, 3-sistosterol, daucosterol and campesterol); nucleotides, and nucleosides, including adenine, adenosine, inosine, cytidine, cytosine, guanine, uridine, thymidine, uracil, hypoxanthine, guanosine, uracil, uridine, guanosine, and deoxyuridine and cordycepia; saturated and unsaturated fatty acids, their derivatives and other organic acids (oleic, linoleic, palmitic and stearic acids); vitamins (B1, B2, B12, E, and K); and inorganic elements (K, Na, Ca, Mg, Fe, Ca, Mn, Zn, Pi, Se, Al, Si, Ni, Si, Ti, Cr, Ga, V, and Zr).	cordycepin, cordycepic acid, pentostatin, carotenoids (lutein, zeaxanthin, cordyxanthins), L-ergothioneine, ergosterol, polysaccharides, glycoproteins, 5-Methyltryptamine Lovastatin 5-Hydroxy-L-tryptophan L-Tryptophan Serotonine L-Phenylalanine

**Table A4:** The pharmacological activity of bioactive compounds of *O. sinensis* and *C. militaris* *).

Classification	Compound, the Pharmacological Activity, and the Reference
*Nucleoside* and *bases*	Adenosine: Neuroprotection, immunomodulatory [266,267] Cordycepin: Neuroprotection, anti-metastatic, anti-platelet aggregation, anti-inflammatory activity, anticancer [62,129,268] Dimethylguanosine: Antioxidant and HIV-1 protease [269] Guanosine: Immunomodulatory [266,267] Cordysinin B: Anti-inflammatory activity [71]
*Sterols*	Ergosteryl-3-O-β-D-glucopyranoside: Anti-inflammatory, antioxidant [71,81] 5α,8α-epidioxy-22E-ergosta-6,9-(11)-22-trien-33β-ol: Cytotoxic against HL-60 cell line [270] 5α,6α-epoxy-5α-ergosta-7,22-dien-3β-ol: Cytotoxic against HL-60 cell line [270] 5α,8α-epidioxy-24(R)-methylcholesta-6,22-dien-3β-Dglucopyranoside: Antitumor [81] Ergosta-4,6,8(14),22-tetraen-3-one: Antitumor [81] 22-dihydro-ergosteryl-3-O-β-D-glucopyranoside: Antitumor [81]
*Cyclodipeptides*	Cordyceamide A: Cytotoxicity against L929, A375 and Hela cell lines [271] Cordyceamide B: Cytotoxicity against L929, A375 and Hela cell lines [271] Cycloaspeptide A: Cytotoxicity against HeLa and MCF7 cell lines [272] Cycloaspeptide C: Cytotoxicity against HeLa and MCF7 cell lines [272] Cycloaspeptide F: Cytotoxicity against HeLa and MCF7 cell lines [272] Cycloaspeptide G: Cytotoxicity against HeLa and MCF7 cell lines [272] Cyclo(L-Pro-L-Val): Antioxidant, anti-inflammatory [71] Cyclo(L-Phe-L-Pro): Antioxidant, anti-inflammatory [71] Cyclo(L-Pro-L-Tyr): Antioxidant, anti-inflammatory [71]
*Alkaloids*	Cordysinin A: Anti-inflammatory, antioxidant [16,19] Cordysinin C: anticancer [16,70] Cordysinin D: anticancer [16,70] Flazin: Antioxidant, anti-inflammatory [70] Perlolyrine (100) Antioxidant, anti-inflammatory, anticancer [70] α-methoxy-α-trifluoro-methylphenylacetyl chlorides: Anti-inflammatory [71] 1-acetyl-β-carboline: Anticancer [70,71] gliocladicillins A: antitumor cell proliferation inhibitors and apoptosis inducers [16,69] gliocladicillin B: antitumor cell proliferation inhibitors and apoptosis inducers [16,69] 11,11′-dideoxyverticillin: antitumor cell proliferation inhibitors and apoptosis inducers [16,69]
*Flavonoids*	3′,4′,7-trihydroxyisoflavone: Antioxidant [71] Diadzein: Antioxidant, anti-inflammatory [71] 6,7,2′,4′,5′-pentamethoxyflavone: Antioxidant activity, anti-HIV-1 protease [269] Glycitein-7-O-β-D-glucoside-4′-O-methylate: Anti-inflammatory [273] Iso-sinensetin (120) Antioxidant activity, anti-HIV-1 protease [269]
*Miscellaneous*	Ophicordin: Antifungal [274] 2-Furancarboxylic acid: Anti-inflammatory, antioxidant [71] 3-hydroxy-2-methyl-4-pyrone: Anti-inflammatory, antioxidant [71] Cordycerebroside A: Anti-inflammatory [275] Soyacerebroside I: Anti-inflammatory [275] Glucocerebroside: Anti-inflammatory [275]

*—Data extracted from the review Olatunji et al., 2018 [19].

**Table A5:** Summary of biomarkers and pharmacological activity used for quality control of Cordyceps [18].

*Nucleosides*	
	Anti-tumor activities; Ca2+ antagonist; depresses the excitability of CNS neurons, inhibits release of various neurotransmitters presynaptically and anticonvulsant activity [72,73,74] stimulate axon growth in vitro and in the adult central nervous system [75]
*Polysaccharides*	
	Anti-oxidation, immuno-potentiation, anti-tumor, and hypoglycemic activity [76,77,78,79]; anti-inflammatory activity and suppresses the humoral immunity in mice [80]
*Ergosterol and its analogs*	
	Cytotoxic activity, anti-viral activity, and anti-arrhythmia effect [81,82]; suppress the activated human mesangial cells and alleviate immunoglobulin A nephropathy (Berger’s disease) [83]
*Mannitol*	
	Diuretic, anti-tussive, and anti-free radical activities [18]
*Peptides*	
	Anti-tumor and immuno-potentiation activities [18]

**Table A6:** Pharmacological activity of total extracts of *Ophiocordyceps sinensis* and *Cordyceps militaris* in experimental study models [19,97].

*O. sinensis*	*C. militaris*
Anti-arteriosclerosis: Rats	Acetylcholinesterase inhibition
Anticancer Mice	Anti-allergic Mice
Anti-diabetic Rats	Anticancer A 4T1, SMMC-7721, BGC-823,MCF-7 cells
Anti-fatigue Rats	Anti-HCV
Anti-fibrotic HK-2, HLFS cells	Anti-HCV
Anti-hypertensive Rats	Antihyperglycemic Mice
Anti-inflammatory HM cells	Antihyperlipidemic Mice
Antioxidant	Anti-inflammatory human ADMSC cells
Anti-thrombotic Humans	Antimicrobial
Antitumor S-180 cells	Anti-obesity C58BL/6 J mice
Hepatoprotective HepG2 cells	Antioxidant
Immunomodulatory RAW 264.7 cells	Antitumor J6/JFH1-huh 7.5 cells
Radio-protective Mice	Hepatoprotective Mice
Renoprotective Rats	HIV-1 protease inhibiting
	HuH-7-derived OR6, AH1R cells
	Hypouricemic Mice
	Immunomodulatory Sea Cucumbers

**Table A7:** Dietary uses of *O. sinensis* in medicinal dishes.

Dishes	Indications for Use
Cooked with an old duck	For patients with cancer, asthenia, or after severe illness
Cooked with hen	For hyposexuality (especially emission)
Cooked with black-bone hen	For asthenia (especially Qi-Yin asthenia)
Cooked with lean pork	For fatigue, male impotence, and “kidney” asthenia
Cooked with sparrow	For antiaging/senescence
Cooked with quail	For fatigue, poor appetite, “kidney” asthenia, and tuberculosis
Cooked with steamed turtle	For male/female hyposexuality
Cooked with baked abalone	For chronic bronchitis, COPD, tuberculosis, arteriosclerosis, cataracts, and for healthy individuals in any season

Data adapted from Jiang (1994) [135] and Zhu et al. (1998) [13].

### Appendix A.3. Clinical Studies

**Table A8:** Summary of Randomized Placebo-Controlled Clinical Trials of *O. sinensis* and *C. militaris*.

Species	Botanical Material/Product	Comparator	Daily Dose	Duration	Population/Condition	Sample Size	Study Design (GCP) *	Primary Endpoints	Outcome Measures	Key Clinical Results	Effect Size (reported/Calculable)	Risk of Bias (Cochrane)	Citation
*O. sinensis*	Cs-4 mycelium (Paecilomyces hepiali)	Placebo	999 mg/day (333 mg TID)	12 weeks	Healthy elderly (50–75 y)	n = 20	DB-PC RCT	Exercise performance	VO_2_max, ventilatory threshold, VT, MT	↑ ventilatory & metabolic thresholds; no VO_2_max change	VO_2_max SMD ≈ 0	Low	Chen et al., 2010 [150]
*O. sinensis*	Mycelium extract	Placebo	2 g/day	12 weeks	Amateur marathoners	n = 12	DB-PC RCT	Aerobic performance	CPET, HR	↓ HR at submax load; ↑ aerobic performance	SMD small–moderate	Moderate	Savioli et al., 2022 [159]
*O. sinensis*	Cs-4	Placebo	3 g/day	12 weeks	Healthy elderly	n = 37	Randomized DB-PC	Aerobic capacity	VO_2_max, FEV_1_	Significant ↑ VO_2_max	SMD ≈ 0.54	Moderate	Xiao et al., 2004 [158]
*O. sinensis*	Cs-4 (CordyMax)	Placebo	3 g/day	5 weeks	Trained cyclists	n = 25	Randomized DB-PC	Endurance	Time-to-exhaustion	No difference	SMD ≈ 0.00	Low	Parcell et al., 2004 [156]
*O. sinensis*	Herbal formulation	Standard asthma care	NR	3 months	Moderate–severe asthma	n = 120	RCT	QoL	AQLQ, FEV_1_	Significant QoL improvement	MD (AQLQ) +0.8	Moderate	Wang et al., 2016 [165]
*O. sinensis*	Cs-4 mycelium, capsule	Placebo “Wait-list group.”	1.6 g (4 caps 0.4 g/caps daily)	12 weeks, follow-up at week 24	Long COVID	n = 110 (55/55}	Waitlist-controlled RCT	Change in the symptom severity, COVID-19 Yorkshire Rehabilitation Scale (C19-YRSm **) at 12 weeks	Long Covid symptoms severity (C19-YRSm&), Fatigue, sleep, QoL, depression, anxiety,	Significant multi-domain improvement. Improved Long COVID severity symptoms, fatigue & QoL	Moderate, Significant change from baseline between-group difference (adjusted for duration and vaccine doses)	Low Some concerns	Chen et al., 2025 [163]
*O. sinensis*	Mycelium extract	Standard care	NR	≥8 weeks	Diabetic kidney disease	>13,000 (meta-analysis)	Systematic review of RCTs	Renal surrogates	Proteinuria, Cr	↓ proteinuria, ↓ Cr	Pooled MD significant	High	Xue et al., 2024 [140]
*O. sinensis*	Mycelium extract	Standard care	NR	NR	Dialysis patients	n = 2914	Meta-analysis	Inflammation, anemia	CRP, Hb, Alb	Improvements in surrogates	Low certainty	High	Liu et al., 2024 [136]
*O. sinensis*	Mycelium extract	Placebo	NR	Peri-procedure	CA-AKI risk	n = 1271	Systematic review	AKI incidence	Serum Cr	↓ CA-AKI incidence	RR <1	Moderate	Pu et al., 2024 [138]
*O. sinensis*	Mycelium	Placebo	1 g (3.5 mg of adenosine and 40 mg polysaccharide)	Single dose	Healthy young adults	n = 14	DB crossover RCT	Muscle recovery	CD34^+^/Pax7^+^ cells	Accelerated stem-cell recruitment	Not pooled	Low	Dewi et al., 2024 [276]
*O. sinensis*	Cordyceps capsules	Placebo	1.5 g (3 × 500 mg)	15 days	Mild-moderate COVID-19	65 (32/33)	Double-blind RCT	Recovery time	Viral load days at hospital; Serum biomarkers	Faster recovery vs placebo	Moderate	Some concerns	ANM Health (2023) [164]
*O. sinensis*	*Cordyceps sinensis*	Chemo alone		6 months	NSCLC	60	RCT		Survival, QoL	Improved survival, QoL	Moderate	Some concerns	Hao et al. (2008) [277]
*O. sinensis*	Cordyceps + NP regimen	NP regimen		6 months	Advanced NSCLC	80	RCT		Tumour response	Improved response rate	Moderate	Some concerns	Hao et al. (2007) [278]
*O. sinensis*	CBG-CS-2 mycelium	Placebo		8 weeks	Healthy adults	80	Double-blind RCT		Immune markers	↑ immune markers	Moderate	Low	Jung et al. (2019) [279]
*O. sinensis*	Cordyceps + anthocyanin	Placebo		8 weeks	Middle-aged adults	40	Double-blind RCT		Mental condition	Improved mood	Small	Some concerns	Morikubo et al. (2005) [280]
*O. sinensis*	Cordyceps extract	Placebo		6 weeks	Asthma	120	Double-blind RCT		HRQoL	Improved HRQoL	Moderate	Some concerns	Wang et al. (2016) [165]
*O. sinensis*	*C. sinensis* capsules	Placebo	2400 mg in 6 capsules	8 weeks	Healthy young adults	30	Double-blind RCT		Testosterone, strength	No effect	None	Low	Hsu et al. (2011) [155]
*O. sinensis*	Extract powder	Placebo	0.5 g	2 weeks	Exhaustive running exercise	36	Double-blind RCT	Recovery time	Respiratory variables, heart rate, and lactate,	↓ fatigue ↓ recovery time	Significant difference vs. placebo	Some concerns	Nagata et al., 2006 [152]
*C. militaris*	*C. militaris*	Placebo		3 weeks	Healthy adults	28	Double-blind RCT		Exercise tolerance	↑ tolerance	Small	Low	Hirsch et al. (2017) [153]
*C. militaris*	Fruiting body extract (standardised)	Placebo	NR	8 weeks	Healthy adults	n = 40	DB-PC RCT	Immune response	NK activity	Significant ↑ NK activity	SMD moderate	Low	Ontawong et al., 2024 [162]
*C. militaris*	Mycelium extract	Placebo	1.5 g/day	8 weeks	Mild liver dysfunction	n = 57	Randomized DB-PC	Liver enzymes	ALT, AST	Significant ↓ ALT & AST vs. placebo	MD −12 U/L Moderate	Low	Heo et al., 2015 [166]
*C. militaris (dominant)*	PeakO_2_ mushroom blend	Placebo	1.0–2.0 g/day) and 12 g/day	28 days	Healthy adults	n = 40 + 43	DB-PC RCT	Endurance	VO_2_peak, Time-to-fatigue	↑ VO_2_peak in subgroups: Improved tolerance vs. baseline	ES not calculable (no control SD), Heterogeneous	Moderate	Dudgeon et al., 2018 [161]
*C. militaris*	Fruiting body extract	Placebo	1.8 g/day	16 weeks	Endurance athletes	n = 11	DB-PC RCT	Hematology	Hb, CK	↑ Hb, ↓ muscle damage	MD clinically relevant	Low	Nakamura et al., 2024 [154]
*C. militaris*	Mycelium capsules+ duloxetine	Placebo + duloxetine	4 g/day	6 weeks	Depression with insomnia	n = 59	Double-blind RCT	Changes in the Athens Insomnia Scale (AIS) score Sleep quality	Sleep quality PSQI, ISI	*C. militaris* did not improve sleep symptoms in patients with depression; no superiority over placebo.	0.6	Low–Moderate	Zhou et al., 2021 [167]

*—DB-PC = double-blind placebo-controlled, RCT = randomized controlled trial, ES = effect size, RoB = Risk of Bias (Cochrane domains, summary judgment); **—C19-YRSm—modified COVID-19 Yorkshire Rehabilitation Scale including the following symptoms in the symptom severity subscale: breathlessness; cough/throat sensitivity/voice change; fatigue; smell/taste; pain/discomfort; cognition; palpitations/dizziness; post-exertional malaise; anxiety/mood; and sleep. Each item was scored on a scale from 0–3 to indicate severity.

**Table A9:** Clinical trials evaluating *O. sinensis* for fatigue and related outcomes in COVID-19 and Long COVID.

Study, Year	Preparation/Dose	Population	Study Design	Duration	Fatigue-Related Outcomes	Meta-Analysis Results	Main Findings	Key limitations	GRADE-Style Certainty *
Chen et al., 2025 [163]	*O. sinensis* Cs-4^®^ (fermented mycelium)	Long COVID patients	Randomized, waitlist-controlled clinical trial	12 weeks	Fatigue severity scales, exercise tolerance, HRQoL	No meta-analysis available (single RCT); aligns with pooled post-viral fatigue effects of adaptogens	Significant reduction in fatigue severity and improvement in functional capacity and QoL compared with usual care	Single-region study; short follow-up; patient-reported outcomes	Moderate (exploratory) ⬤⬤⬤◯
ANM Health, 2023 [164]	*O. sinensis* Capsules (add-on therapy)	Mild–moderate COVID-19 patients	Randomized, double-blind, placebo-controlled	14–28 days	Fatigue, recovery time, QoL	Not pooled; excluded from formal meta-analyses due to reporting limitations	Faster symptom resolution and reduced fatigue compared with placebo	Industry-sponsored; non–peer-reviewed; limited methodological transparency	Low ⬤⬤◯◯

* Level of evidence reflects EMA/HMPC-oriented qualitative grading, not formal regulatory classification. Abbreviations: HRQoL, health-related quality of life. DB-RCT, double-blind randomized controlled trial.

GRADE-Style Certainty Explanation (Adapted for Frontiers):

- ⬤⬤⬤⬤ **High:** Very unlikely to change confidence
- ⬤⬤⬤◯ **Moderate:** Likely to have an important impact on confidence
- ⬤⬤◯◯ **Low:** Further research is very likely to change the estimate
- ⬤◯◯◯ **Very low:** Evidence uncertainty

### Appendix A.4. Network Pharmacology/Systems Pharmacology

The 22 studies in which network pharmacology/systems pharmacology was combined with wet-lab or clinical validation (animal models, cell experiments, or RCTs) are listed in Table A10. The 15 in-silico prediction studies by network pharmacology analysis are listed in Table A11.

**Table A10:** Overview of network-pharmacology studies of *O. sinensis*, *C. militaris* preparations, and cordycepin, supported by experimental validation.

Study (Year)	Dose/Concentration Main Bioactive Compounds	Disease or Physiological Function	Key Targets/Nodes Validated	Signaling Pathways	Type of Validation & Main Mechanistic Outcome
Jiang et al., 2023 [175]	*O. sinensis*; arachidonic acid, lysergol, glycitein; stigmasterol, sitosterol, linoleic acetate, karanjin, aurantiamide acetate, and berberine. 1000 mg/kg in rats,	Ischemic stroke	CASP3, PTGS2, and PPARG, AR, NOS2, PTGS2, PTGS1, CYP17A1, ADRB2, CHRM2, ESR1, RXRA, and SCN5A	IL-17, AGE-RAGE, and TNF signaling pathways	The mechanism of action of *O. sinensis* is related to the regulation of blood lipids, anti-apoptotic effects, and anti-inflammatory effects. The results of the docking analysis suggest that sitosterol, lysergol, and stigmasterol have high affinities to some core proteins (CASP3, PTGS2, PPARG, JUN, and ESR1.
Wang et al., 2017 [177]	*O. sinensis*, Cordycepin. 0.2, 1 or 2 mg/kg In mice	Hepatitis B vaccine adjuvant	Immune-response targets related to B- and T-cell activation and cytokine signaling	Immune & vaccine-response pathways (e.g., T-cell receptor, cytokine signaling)	Systems pharmacology predicted cordycepin as an adjuvant; BALB/c mouse vaccination showed enhanced humoral and cellular HBV responses without apparent toxicity. Effects of cordycepin (C) on serum HBV antibodies, lymphocyte proliferation and cytokine levels in spleen cell supernatants.
Zhang X et al., 2023 [178]	*O. sinensis* extract A total of 54 active ingredients which are not specified for their name and chemical structure. 300, 600, 1200 mg/kg in mice	Lung adenocarcinoma	Breast cancer type 1 susceptibility protein (BRCA1) and G1/S-specific cyclin-E1 (CCNE1) and oxidative-stress regulators	PI3K–Akt, HIF-1 signaling, apoptosis pathways	Bioinformatics + network analysis predicted anti-Lung adenocarcinoma targets; in vivo validation showed *O. sinensis* inhibits tumor growth and modulates these pathways.
Ma and Jin, 2024 [143]	*O. sinensis* 121 bioactive compounds, including cordycepin	Chronic obstructive pulmonary disease (COPD)	CXCR4, PDGFRB, PARP1, SRC, HIF1A, NFKB1, HDAC 2, and PKACA	Chemokine signaling	The target analysis of the Bailing capsule’s effects revealed interactions with multiple targets. The study suggested that the mechanism might involve interactions with chemokines, tyrosine kinase receptors, and other related molecular signaling pathways.
Zhou et al., 2025 [174]	*C. sinensis* Sphingolipid. 0.405 g/kg/day (L-CS), 0.81 g/kg/day (M-CS), and 1.62 g/kg/day (H-CS); in rats	Chronic obstructive pulmonary disease (COPD)	PLA2G4E and B4GALT4 proteins AKT1, ESR1, TLR4, and MMP9; TNF-α, IL-8, and multiple metabolic proteins	PI3K-AKT signaling pathway Inflammatory and metabolic pathways	*O. sinensis* alleviated lung injury, cytokine profiles, and inflammation in the COPD model of rats; Proteomics + metabolomics + network analysis connected altered proteins/metabolites to COPD-relevant pathways including PI3K-AKT signaling pathway in COPD rats, potentially affecting glycerophospholipid metabolism and sphingolipid metabolism by targeting PLA2G4E and B4GALT4 proteins, thereby alleviating the inflammatory response and mitigating lung tissue damage caused by COPD.
Zhang Y et al., 2023 [179]	*O. sinensis*; arachidonic acid, linoleyl acetate, cerevisterol, beta-sitosterol, peroxyergosterol, cholesterol, and cholesteryl palmitate. 50 μg/mL fermented *O. sinensis* (in vitro a proximal tubular HK-2 cell line derived from normal human kidney)	diabetic kidney disease	RELA, JNK1, PTEN, VEGFA, EGF, ERK2, CASP3, AKT1, MMP9. *O. synensis* downregulated the expressions of Bax, Caspase-3, VEGFA, P-AKT, and P-ERK, and upregulated the expression of PTEN	AKT and ERK signaling pathway	*O. synensis* has nephroprotective effects, which functions via promoting proliferation and inhibiting apoptosis of renal proximal tubular cells, likely by targeting Caspase-3, Bax, VEGFA and PTEN.
Li et al., 2024 [180]	*O. sinensis* adenosine; 2′deoxyadenosine; cordycepin; adenine; uracil; hypoxanthine; uridine; guanosine hydrate; thymidine. CS extract (150 mg/kg in mice; 2′-deoxyadenosine The dose is Not specified	acute kidney injury (AKI)	STING, Irf3, Perforin, IFN-γ, GAPDH	STING/IRF3 pathway	2′-deoxyadenosine treatment significantly alleviated FA-induced renal damage in vivo and alleviated the renal injury in NK cells by activating the STING/IRF3 pathway to inhibit perforin release in vitro. 2′-deoxyadenosine could mitigate AKI by downregulating NK cell activity (by decreasing perforin and IFN-γ expression) and inhibiting the stimulator of interferon genes and phosphorylated IFN regulatory factor 3.
Tao et al., 2024 [139]	*O. sinensis* mycelial preparation (Bailing capsules), including arachidonic acid, linoleoyl acetate, cerevisterol, beta-sitosterol, peroxyergosterol, cholesteryl palmitate, and cholesterol 0.8–5 g/day in humans	Chronic kidney disease (CKD), human RCT	Inflammatory & metabolic renal function markers including: PTPN1, HSD11B1, HSD11B2, HMGCR, AR, NR1H3, NR3C1, CNR2, CYP19A1, CYP17A1, and DRD2	Neuroactive ligand-receptor interaction, Chemical carcinogenesis receptor activation, Diabetic cardio-myopathy, cAMP signaling, Inflammatory mediator regulation of TRP channels, Insulin resistance, Proteoglycans, Serotonergic synapse, AGE-RAGE signaling, EGFR tyrosine kinase inhibitor resistance, Prolactin signaling, Endocrine resistance, C-type lectin receptor, ErbB, VEGF, Arachidonic acid metabolism, and Adipocytokine signaling pathways.	Network pharmacology analysis identified 190 common targets of *O. synensis* (Bailing Capsule) and chronic kidney disease associated with immune response, inflammatory response, vascular endothelial damage, cell proliferation, and fibrosis. Clinical trials showed improved renal indices and reduced inflammation when the Bailing capsule was added to standard CKD therapy. Mechanistic validation is indirect but consistent.
He et al., 2020 [181]	*O. sinensis* Adenosine exopolysaccharide, amino acid, mannitol human keratinocyte line HaCaT (50, 250, and 500 μg/mL extract	UVB-induced damage in human keratinocytes	Aquaporin 3 (AQP3)	PPAR signaling pathway, cholesterol metabolism, and ovarian steroidogenesis.	Cordyceps significantly decreased intracellular UVB-induced oxidative stress, including ROS production and intracellular H2O2 content. Besides, AQP3, which mediates intracellular signaling and transports H2O2 into cells, was significantly increased in the presence of Cordyceps extract under UVB irradiation. In addition, the DNA repair effect of Cordyceps extract after UV irradiation was proven to be effective by the comet assay.
Pei et al., 2023 [182]	*C. militaris* polysaccharides	Immune modulation (non-specific, innate/adaptive immunity)	TNF, MAPK3, CASP3, VEGFA, STAT3; TLR4	Toll-like receptor (TLR4), TNF-α signaling; macrophage M1↔M2 polarization and immune activation, apoptosis, cytokine pathways	Network analysis identified immune targets enriched in TLR pathways; in vitro macrophage assays showed altered polarization, and in vivo mouse experiments confirmed TLR4/TNF-α activation by *C. militaris* polysaccharides consistent with predictions.
Kim et al., 2025 [183]	*C. militaris* extracts (cordycepin, adenosine) Lung cancer cell lines (LLC1, H460, H1299	Lung cancer	p53, EGFR, apoptosis-related targets	PI3K–Akt, p53, apoptosis	Network pharmacology/docking indicated cordycepin and adenosine as key ligands targeting EGFR/p53-related networks; cell experiments showed enhanced apoptosis and anticancer activity, particularly after optimizing extraction/drying procedures to enrich key actives.
Wang et al., 2025 [184]	*C. militaris* fruiting body; nucleosides (uridine, guanosine, adenosine, cordycepin, N^6^-(2-hydroxyethyl)adenosine);	Chronic obstructive pulmonary disease (COPD)	IDH1, CYP19A1, lipid-metabolism targets	Linoleic acid metabolism, inflammatory and oxidative pathways	HPLC fingerprinting + metabolomics + network pharmacology; COPD mouse model demonstrated improved lung function and pathology; docking supported strong binding of CM components to IDH1/CYP19A1.
Zhou et al., 2024 [185]	*C. militaris* (cordycepin, ergosterol) 100 and 200 mg/kg/d *C. militaris* extract;	Gouty nephropathy/hyperuricemia	cyclooxygenase-2 (COX-2); renal transport proteins ABCG2, GLUT9, and URAT1; Xanthine oxidase (XO),	Inflammation (PTGS2, NLRP3, etc.) signaling pathway and uric acid metabolic pathway (XDH, ADA, UMOD).	Network pharmacology suggested cordycepin + ergosterol as core ligands; in vivo data show anti-hyperuricemic effect via XO inhibition and modulation of renal urate transporters.
Gandhale et al., 2024 [186]	*C. militaris* adenosine, cinnamic acid, citric acid, cordycepin, dipicolinic acid, ergosterol, fumaric acid, hypoxanthine, N-acetylgalactosamine, p-hydroxybenzoic acid, β-sitosterol, and δ-tocopherol Vero E6 cells (25, 50, 75, 100 μg/mL) *C. militaris* aqueous extract in-vitro	SARS-CoV-2	Multiple targets	Numerous interactions	Cordycepin, Cicadapeptin-I, Cicadapeptin-II, Cordycerebroside-B, and N-Acetyl galactosamine were found to be top scorers To assess the anti-SARS-CoV-2 activity of the *C. militaris* aqueous extract in-vitro, at 80% confluency, Vero E6 cells were infected in triplicate with SARS-CoV-2
Lee et al., 2019 [187]	Cordycepin and *C. militaris* extract constituents MCF-7 human breast cancer cell line, treatment with *C. militaris* (100 µg/mL) and cordycepin (25, 50 µM).	Breast cancer (MCF-7 cells)	Apoptosis-related proteins: CASP3, BAX, BCL2, X-linked inhibitor of apoptosis protein (XIAP) Caspases; p53, Hedgehog signaling components	Apoptosis, p53, Hedgehog, estrogen, PI3K–Akt, pathways	Cordycepin exhibited the ability to induce apoptotic cell death by increasing the cleavage of caspase-7, -8, and -9, increasing the Bax/Bcl-2 protein expression ratio, and decreasing the protein expression of X-linked inhibitor of apoptosis protein (XIAP) in MCF-7 cells. Consequently, the *C. militaris* concentrate and cordycepin exhibited significant anticancer effects through their ability to induce apoptosis in breast cancer cells.
Chen et al., 2024 [57]	Cordycepin (10 μM, 20 μM, 50 μM, 100 μM, or 200 μM) in The human breast cancer cell lines MCF7 and MDA-MB-231, as well as the human monocyte cell line THP-1.	breast cancer	Alumin gene ALB	protein tetramerization, regulation of protein complex disassembly, and somatic diversification of immunoglobulin signaling	Cordycepin regulates tumor immune suppression by upregulating the downregulated ALB, thereby playing an anti-tumor role.
Qui et al., 2025 [188]	Cordycepin	Fibrosarcoma	AKT1	Akt1 (protein kinase B) and disruption of protein phosphorylation pathways	Cordycepin significantly inhibited cell activity at an effective concentration of 100 μmol/L. Key observations included changes in cell morphology, reduced migration, inhibited proliferation, cell cycle arrest at the G0/G1 and G2/M phases, and induction of apoptosis. Western blot analysis further confirmed that cordycepin simultaneously downregulated both the expression and phosphorylation levels of Akt in a dose-dependent manner.
Zhong et al., 2025 [189]	Cordycepin 50 mg/kg/d, 100 mg/kg/d, 200 mg/kg/d of cordycepin in mice	Cancer immunosuppression	EGFR, upregulated the protein expression of Nrf2, NQO1 and HO-1 in the spleens	metabolic and immune pathways	Cordycepin ameliorated cyclophosphamide-induced immunosuppression of mice by reversing metabolic dysfunction and activating the Nrf2 pathway through regulating EGFR, indicating its potential as a therapeutic agent for immunosuppression.
Chen et al., 2022 [190]	Cordycepin (alone and in combination) 80 mM of cordycepin in Human glioblastoma cells (LN-229, U251, T98G)	Glioblastoma (GBM)	Multiple tumor and apoptosis targets (predicted network)	PI3K-Akt, apoptosis, cell proliferation pathways	Network pharmacology predicted synergy; in vitro assays supported enhanced cytotoxicity when cordycepin combined with doxorubicin; docking suggested binding to targets involved in GBM survival.
Wang et al., 2025 [102]	Cordycepin 20 mg/kg and 40 mg/kg In rats	Chronic unpredictable mild stress (CUMS) induced depression	Cordycepin increased protein levels of p-GSK3β, β-catenin, and nuclear β-catenin, and enhanced transcription of downstream genes PKM, LDHA, Cyclin D1 and C-myc in brains of CUMS-induced rats	GSK3β/β-catenin signaling	Cordycepin exerted an antidepressant effect by modulating the GSK3β/β-catenin pathway. Western blot and Real-time PCR were applied to validate the signaling pathway.
Lin et al., 2024 [191]	Cordycepin	Pulmonary arterial hypertension (PAH)	TP53, AKT1, CASP3, BAX, BCL2L1	Apoptosis, PI3K–Akt, vascular remodeling pathways	Network analysis and docking identified PAH-core genes; in MCT-induced PAH rats, cordycepin reduced RVSP and vascular remodeling and modulated PASMC proliferation/apoptosis,
Liao et al., 2025 [192]	Cordycepin (±5′-monophosphate) 40 mg/kg in mice.	Western-diet–induced obesity	CPS1, HRAS, MAPK14, AKT1, GSK3B, EGFR, CASP3, APOA1/2/3, APOM, etc.	Metabolic pathways, insulin signaling, HIF-1, FOXO, lipid & atherosclerosis, TNF, IL-17, Toll-like receptor signaling, inflammatory pathways	Integrated network pharmacology, transcriptomics, and docking analysis identified 244 potential targets and core hubs; cordycepin improved obesity and metabolic parameters in animal experiments: core targets and pathways involved in obesity were validated by gene expression and phenotypic changes. The authors uncover a potential mechanism of action of cordycepin against obesity through network pharmacology and quantitative transcriptomics, providing evidence for obesity pathogenesis and suggesting that cordycepin is a potential lead compound for anti-obesity treatment.

**Table A11:** Overview of network-pharmacology studies on *Ophiocordyceps sinensis*, *Cordyceps militaris*, and cordycepin, in-silico predictions without experimental validation.

Study (Year)	Main Bioactive Compounds	Disease or Physiological Function	Key Targets/Nodes Validated	Signaling Pathways	Type of Mechanistic Outcome
Li J. et al., 2021 [193]	*O. sinensis*, 6 main active ingredients, including adenosine, ergosterol,	Liver cirrhosis,	MAPK1, CASP8, TNF, VEGFA	Hepatitis B, cancer, apoptosis, and inflammation signaling	Modulation of inflammatory and apoptotic pathways.
Mu et al., 2023 [194]	*O. sinensis*; arachidonic acid, sitosterol, berberine, Higenamine, Cordycepin, Uralene. Crachidonic aci Caffeine	Liver cancer	TNF, CASP3, BCL2, IL6, VEGF-A, NF, Caspase 3 (CASP3), B-Cell Lymphoma 2 (BCL2), Interleukin-6 (IL-6), Vascular Endothelial Growth Factor-A (VEGF-A), and Prostaglandin-endoperoxide Synthase 2 (PTGS2)	hepatitis B, cancer, Advanced Glycation Endproducts-Receptor for Advanced Glycation Endproducts (AGE-RAGE) signaling pathway, non-alcoholic fatty liver disease, hepatitis C, alcoholic liver disease, and IL-17 signaling pathway, Nucleotide-binding Oligomerization Domain (NOD)-like receptor signaling pathway, and TNF signaling pathway	The authors suggest that the antitumor effect of *O. sinensis* primarily in hepatocellular carcinoma originates from its intricate influence on the target proteins through a complex interplay of its constituents.
Gonzalez-Llerena et al., 2025 [195]	129 compounds, including cordycepsidone A, jiangxienone, and flazin, exhibiting binding affinity comparable or superior to clinically used inhibitors across the Cordyceps genus (including *C. militaris*, *O. sinensis*)	Cancer (broad anticancer potential)	Hub proteins such as TYMS, AURKA, and CDK1 were identified as primary targets, Multi-target lists across cancer biology (eg. EGFR, AKT, CASP families)	PI3K-Akt, MAPK, apoptosis, cell cycle, immune pathways; Oncogenic pathways, including cell cycle regulation, DNA replication, and apoptosis.	Systematic identification of putative multitarget anticancer agents in Cordyceps genus; suggests prioritized compounds for further testing.
Ma et al., 2022 [60]	*O. sinensis*, adenosine, ergosterol, nucleosides	Oral lichen planus (OLP)	TNF, IL6, CD4, EGFR, IL1B	PI3K–Akt, MAPK, apoptosis, T-cell activation	Predicted multi-target action; docking supported ligand-target interactions; authors propose anti-inflammatory/immunomodulatory mechanism.
Long et al., 2021 [196]	*O. sinensis* Including daucosterol, vitamin A, inosine, peroxyergosterol, vitamin B1, cerevisterol, linoleic acid, alpha-trehalose, and galactomannan	Hypoxia	O. cordyceps inctrease the expression of MAPK1,MAPK3, VEGFA, and decrease the expression of AKT1, PIK3CA, and RAC1 under hypoxic conditions	VEGF signaling pathway	*O. sinensis* promotes angiogenesis by regulating the VEGF signaling pathway, which might be one of the mechanisms of hypoxia adaptation, and improves the survival rate of H9C2 cells.
Zhang et al., 2022 [197]	*O. sinensis*, 7 main active ingredients: arachidonic acid, linoleyl acetate, beta-sitosterol, peroxyergosterol, cerevisterol, cholesteryl palmitate, and cholesterol	Depression Antidepressant, serotonergic, Anti-neuroinflammatory	Catalase (CAT), CREB binding protein (CREBBP), epidermal growth factor (EGF), and E1A binding protein P300.	The FOXO, the hypoxia-inducible factor 1 (HIF-1), and Huntington’s disease signaling pathways, oxidative stress response, and neurotrophic signaling	Authors propose antioxidant and epigenetic (CREB/EP300) modulation as potential antidepressant mechanisms; docking supports several compound–target interactions.
Zhou et al., 2023 [198]	*O. sinensis* D-glutamine 2,3-dihydroxypropyl hexadecanoate 4-(2-aminopropyl) − 2-methoxyphenol Caffeine	Influenza infection	SRC, RHOA, HSP90AA1, VEGFA, EGFR	PI3K-Akt, HIF-1, Influenza A, COVID-19 Innate immunity, cytokine signaling, and antiviral response. Butanoate, thiamine, amino-acid metabolism, TCA, arginine biosynthesis	Predicted immunomodulatory and antiviral host-target modulation and maintain respiratory immune balance via NF κB/IL-17 signaling and PI3K–AKT–linked survival pathways.; docking supported interactions with immune signaling proteins. UPLC-MS metabolomics + network pharmacology + molecular pharmacology; differential metabolites mapped to antiviral pathways, and pharmacological assays supported the anti-influenza effects of selected components by network mapping and glutamine docking to SRC and EGFR, targeting viral/airway targets.
Li Y. et al., 2021a [199]	*O. sinensis*, Seven active ingredients including arachidonic acid, linoleyl acetate, cerevisterol, beta-sitosterol, peroxyergosterol, cholesterol, and cholesteryl palmitate	Diabetic nephropathy (DN)	TNF, MAPK1, EGFR, ACE, CASP3	AGE-RAGE signaling pathway in diabetic complications, TNF signaling pathway, PI3K-Akt signaling pathway, and IL-17 signaling pathway	The mechanism of multicomponent, multitarget, and multichannel action of *O. sinensis* in treating diabetic nephropathy is due to targeting TNF, MAPK1, EGFR, ACE, and CASP3 signaling pathways, which are involved in the inflammatory response, apoptosis, oxidative stress, and insulin resistance.
Xi et al., 2024 [200]	Bailing capsules (*O. sinensis* preparations), various metabolites	Diabetic nephropathy	TNF, IL6, TGF-β–related nodes, oxidative stress targets	AGE-RAGE, PI3K–Akt, inflammatory and fibrotic signaling	Network-pharmacology synthesis suggests Bailing capsules act via anti-inflammatory, anti-oxidative and anti-fibrotic multi-target effects in DN.
Guan et al., 2023 [201]	*O. sinensis*; 106 compounds including linoleyl acetate, cholesteryl palmitate, arachidonic acid, and	Polycystic ovary syndrome	JAK2, PPARG, PI3K, and AKT1 were upregulated, whereas those of ESR1 and IRS1 were downregulated in PCOS model mice.	JAK-STAT and PI3K-Akt signaling pathways	qPCR findings indicated that BL exerted anti-PCOS effects via PIK3CA, ESR1, AKT, PPARG, and IRS1 targets affecting PI3K-Akt signaling pathways
Singh et al., 2024 [202]	16 steroids including beta-sitosterol, cholest-5-en-3β-ol, 3β, and 7α-Dihydroxycholest-5-ene Cholest-4-en-3-one	SARS-CoV-2	Glucocorticoid receptor (NR3C1). thrombin (F2),	17 inflammatory pathways	*Cordyceps militaris* as an add-on therapy that may reduce the progression of inflammatory co-morbidities among patients infected with SARS-CoV-
Chen et al., 2025 [203]	Cordycepin	Colorectal Cancer	24 drug targets	activates the p53 signaling	Cordycepin inhibits the proliferation of SW480 cells and suppresses tumor growth by modulating the apoptotic pathway.
Li et al., 2025 [204]	Cordycepin	Cancer	A3 adenosine receptor (A3AR),	MAPK, AMPK, mTOR, and Wnt/*β*-catenin	
Khan and Tania, 2023 [205]	Cordycepin	Cancer	JNK, MAPK, AMPK, PI3K/Akt, ERK, mTOR, GSK-3b, FAK kinases	the c-Jun N-terminal kinase (JNK), mitogen-activated protein kinase (MAPK), AMP kinase (AMPK), phosphoinositide 3-kinase (PI3K)/Akt, extracellular signal-regulated kinase (ERK), mammalian target of rapamycin (mTOR), glycogen synthase kinase (GSK)-3b, and focal adhesion kinase (FAK) pathways	kinase inhibitors can have crucial roles in cancer treatment, targeting tyrosine kinases might be one of the molecular mechanisms involved in the anticancer potential of cordycepin
Ma X. et al., 2022 [206]	Cordycepin	Alzheimer’s disease	AKT1, MAPK8, BCL2L1, FOXO3, and CTNNB1 associated with pathogenic genes APP, MAPT, and PSEN2 and with longevity in Alzheimer’s Disease	PI3K-Akt, MAPK, apoptosis, neuroinflammation pathways. Lipid and atherosclerosis,	Network/docking predicted cordycepin interacts with AD-relevant targets and signaling pathways targeting MAPK8, FOXO3, and CTNNB1, which may have significant clinical and treatment implications. The authors present cordycepin as a multi-target candidate for neurodegeneration (with in silico support).

**Table A12:** Abbreviations of proteins.

Abbreviations	Full Name, and Description
AKT1	Protein Kinase B—regulates survival and metabolism
AMPK	AMP-activated protein kinase (PRKAA1/2/PRKAG1)—cellular energy sensor
BECN1	Beclin-1—core autophagy regulator
CASP1	Caspase 1—cleaves IL-1b/IL-18 and activates pyroptosis
FOXO3	Forkhead Box O3—transcription factor promoting stress resistance
GSDMD	Gasdermin D—effector of pyroptosis
HIF1A	Hypoxia-Inducible Factor 1 Alpha—transcription factor for hypoxia response
HMOX1	Heme Oxygenase 1—cytoprotective enzyme against oxidative stress
KEAP1	Kelch-Like ECH-Associated Protein 1—NRF2 inhibitor
LC3B	Microtubule-Associated Protein 1 Light Chain 3 Beta—autophagosome marker
MAPK1/3	Mitogen-Activated Protein Kinase 1/3—cell proliferation & stress signaling
MAPK14/p38	Mitogen-Activated Protein Kinase 14—controls cytokine response
MAPK8/JNK1	Mitogen-Activated Protein Kinase 8—stress-activated kinase
mTOR	Mechanistic Target of Rapamycin—regulator of growth and autophagy
MYD88	Myeloid Differentiation Primary Response 88—adaptor protein for TLR signaling
NF-kB	Nuclear Factor kappa-light-chain-enhancer of activated B cells—inflammation regulator
NLRP3	NOD-Like Receptor Family Pyrin Domain Containing 3—inflammasome component
NQO1	NAD(P)H Quinone Dehydrogenase 1—detoxification enzyme
NRF2	Nuclear Factor Erythroid 2–Related Factor 2—antioxidant transcription factor
PDK1	Pyruvate Dehydrogenase Kinase 1—reduces oxygen consumption by blocking PDH
PGC-1-α	Peroxisome proliferator-activated receptor gamma coactivator 1-alpha—transcriptional coactivator that regulates the genes involved in energy metabolism.
PI3K	Phosphatidylinositol 3-Kinase—upstream activator of AKT
SIRT1	Sirtuin 1—NAD-dependent deacetylase regulating metabolism and aging
SLC2A1	Glucose Transporter 1—mediates glucose uptake under hypoxia
TLR4	Toll-Like Receptor 4—innate immune sensor for LPS
ULK1	Unc-51 Like Autophagy Activating Kinase 1—initiates autophagy
VEGFA	Vascular Endothelial Growth Factor A—angiogenesis mediator

**Table A13:** Cordycepin-induced adaptive stress Response Network signaling pathways and biological functions.

Abbreviation	Full Name	Primary Function
PI3K–AKT/FOXO–SIRT Metabolic Survival Axis		
PIK3CA/PIK3CB	Phosphatidylinositol-4,5-Bisphosphate 3-Kinase Catalytic Subunits	Generate PIP3 for AKT activation.
AKT1	Serine/Threonine Kinase 1	Promotes cell survival and metabolism.
FOXO3	Forkhead Box O3	Transcription factor inducing stress resistance and repair genes.
SIRT1	NAD-Dependent Deacetylase Sirtuin 1	Regulates metabolism, stress resistance, and longevity.
TP53 (p53)	Tumor Protein p53	DNA damage response and apoptosis regulator.
AMPK–mTOR–Autophagy Axis		
AMPK	AMP-activated protein kinase (subunits PRKAA1, PRKAA2, PRKAG1)	Cellular energy sensor; activates catabolic pathways to restore ATP.
mTOR	Mechanistic Target of Rapamycin	Regulates growth, protein synthesis, and autophagy.
ULK1	Unc-51 Like Autophagy Activating Kinase 1	Initiates autophagy signaling downstream of AMPK.
BECN1	Beclin-1	Core autophagy regulator; nucleates autophagosome formation.
LC3B (MAP1LC3B)	Microtubule-Associated Proteins 1A/1B Light Chain 3B	Marker of autophagosome membrane formation.
SQSTM1/p62	Sequestosome 1	Cargo receptor linking ubiquitinated proteins to autophagy machinery.
ATG5, ATG7	Autophagy Related 5 and 7	Essential enzymes for autophagosome membrane elongation.
NRF2–KEAP1 Antioxidant Axis		
NRF2 (NFE2L2)	Nuclear Factor Erythroid 2–Related Factor 2	Transcription factor controlling antioxidant and detoxification genes.
KEAP1	Kelch-Like ECH-Associated Protein 1	Cytoplasmic inhibitor of NRF2; targets it for degradation.
HMOX1 (HO-1)	Heme Oxygenase 1	Degrades heme; produces cytoprotective metabolites.
NQO1	NAD(P)H Quinone Dehydrogenase 1	Detoxifies quinones and prevents redox cycling.
SOD2	Superoxide Dismutase 2, mitochondrial	Converts superoxide radicals to hydrogen peroxide.
CAT	Catalase	Converts hydrogen peroxide to water and oxygen.
GPX1	Glutathione Peroxidase 1	Reduces lipid and hydrogen peroxides using glutathione.
TLR4–MyD88–NF-κB/MAPK Inflammatory Axis		
TLR4	Toll-Like Receptor 4	Pattern-recognition receptor sensing bacterial LPS.
MYD88	Myeloid Differentiation Primary Response 88	Adaptor protein mediating TLR/IL-1 receptor signaling.
IKBKB	Inhibitor of NF-κB Kinase Subunit Beta	Phosphorylates IκB, enabling NF-κB nuclear translocation.
NFKB1/RELA (p50/p65)	Nuclear Factor κB Subunits	Master regulators of inflammation, immunity, and apoptosis.
MAPK1/3 (ERK1/2)	Mitogen-Activated Protein Kinases 1/3	Regulate cell proliferation, inflammation, and stress responses.
MAPK8 (JNK1)	c-Jun N-terminal Kinase 1	Mediates apoptosis and stress signaling.
MAPK14 (p38 MAPK)	Mitogen-Activated Protein Kinase 14	Controls cytokine production and inflammatory response.
TNF	Tumor Necrosis Factor Alpha	Proinflammatory cytokine.
IL6	Interleukin 6	Cytokine linking inflammation to metabolism and stress.
PTGS2 (COX-2)	Prostaglandin-Endoperoxide Synthase 2	Catalyzes prostaglandin synthesis in inflammation.
NOS2 (iNOS)	Inducible Nitric Oxide Synthase	Produces nitric oxide in immune defense and inflammation.
NLRP3 Inflammasome/Pyroptosis		
NLRP3	NOD-Like Receptor Family Pyrin Domain Containing 3	Sensor forming inflammasome complexes in response to stress.
CASP1	Caspase 1	Cleaves pro–IL-1β/IL-18 and activates pyroptosis.
GSDMD	Gasdermin D	Executes pyroptotic cell death upon cleavage.
IL1B	Interleukin 1 Beta	Proinflammatory cytokine processed by inflammasome.
IL18	Interleukin 18	Enhances immune and inflammatory responses.
HIF-1 Hypoxia Response		
HIF1A	Hypoxia-Inducible Factor 1 Alpha	Master regulator of hypoxia responses and metabolic adaptation.
VEGFA	Vascular Endothelial Growth Factor A	Promotes angiogenesis and oxygen delivery.

**Table A14:** Evidence-Tier Framework for Mechanistic Integration.

Evidence Tier	Definition	Data Source	Strength	Limitation
Tier 1: In Silico Prediction	Targets/pathways identified by network pharmacology, molecular docking, and enrichment analysis	KEGG/GO enrichment, PPI networks	Hypothesis-generating	Risk of target inflation; no biological confirmation
Tier 2: Experimental Validation	Predicted pathways confirmed in vitro or in vivo	Cell models, animal studies	Mechanistic support	Often, supra-physiological concentrations, species differences
Their 3: Clinical Evidence	Mechanisms aligned with RCT endpoints or biomarker modulation in humans	RCTs, meta-analyses	Highest relevance	Heterogeneity of preparations; surrogate endpoints

### Appendix A.5. Food and Drug Regulation of Cordyceps Mushroom

**Table A15:** Regulatory status of *O. sinensis* and *C. militaris* worldwide.

Region/Jurisdiction	*O. sinensis*	*C. militaris*	Notes/Caveats/References
China (as food/health-food regulation)	*O. sinensis* is historically used as a medicinal fungus; in China, the wild form is not a regular food, but specific cultivated strains/preparations may have food or “medicine-food homologous” (药食同源) status in some local jurisdictions	*C. militaris* is approved in China as a “new resource food” (新资源食品) (i.e., allowed for food/health food use) since 2009	The Chinese “new resource food” system permits certain traditionally medicinal fungi and their derivatives for official consumption under controlled conditions.
Japan/Korea/East Asia	Often regulated under traditional medicine/health food frameworks, not as general food	*C. militaris* is regarded as an edible and medicinal fungus; considered safe and edible in many Asian settings (used in cuisine and supplements)	In Asian countries, the cultural and historical use supports a more permissive edible status for *C. militaris*.
United States	*O. sinensis* per se is not Generally Recognized As Safe (GRAS)/food-approved; typical marketplace status is as a dietary supplement (mycelium cultures, etc.).	*C. militaris* is generally sold as a dietary supplement; the FDA has issued warning letters when Cordyceps products are marketed as treatment for diseases (i.e., considered new drugs) [233].	The U.S. classifies many mushroom extracts as dietary supplements. If a product claims to treat a disease, the FDA may treat it as a drug.
European Union (Novel Food Regulation)	Mycelium and fruiting body of *O. sinensis* “not novel in food supplements” per updated Novel Food Catalogue (i.e., recognized in supplement use)	*C. militaris* (mycelium and fruiting body) remains classified as unauthorized/novel food in supplements (i.e., not yet accepted)	A February 2025 update clarified that *O. sinensis* mycelium and fruiting bodies are not novel (for supplements), but *C. militaris* is still subject to novel food approval.
EU Food Use beyond supplements	Use of *O. sinensis* outside of supplements (e.g., in foods, beverages) may be treated as Novel and require pre-market authorization under Regulation (EU) 2015/2283	For *C. militaris*, any use (mycelium, fruiting body) in foods or supplements is under novel food control until approved	The non-novel status currently applies only to “food supplements” of *O. sinensis*; other forms may still be novel.

Data derived from peer-reviewed study (Chen 2013 [16]), official food and drug regulatory websites, and Rapid Alert System for Food and Feed (RASFF) notifications [225,226,227,228,229,230,231,232,233,234,235]. The RASFF page shows the alert and the product name. This is robust evidence that *C. militaris* is being flagged by EU member authorities as unauthorized in supplements.

Table A16, Table A17, Table A18, Table A19 and Table A20 were generated by artificial intelligence (AI) technology (ChatGPT for IOS, version 1202, model, 2025 OpenAI, L.L.C.)

**Table A16:** Characteristic feature of *Ophiocordyceps sinensis* and *Cordyceps militaris* across key pharmacopoeias and regulatory frameworks.

*O. sinensis*	*C. militaris*
Chinese Pharmacopoeia (ChP) ◦ ✅ Official monograph for wild *O. sinensis*. ◦ Quality markers include adenosine, polysaccharides, and mannitol. Adenosine is considered a key quality indicator. ◦ Cordycepin is present only in trace amounts in wild specimens (~0.006–0.075 mg/g), so not a standard assay marker. Korean Pharmacopoeia (KP) ◦ ✅ Official monograph for *C. sinensis*. ◦ Standards emphasize adenosine and, likely, polysaccharide content. Vietnamese & Thai Pharmacopoeias ◦ ✅ Often include *C. sinensis* (limited detail available). ◦ Typically regulated as herbal supplements or medicinal fungi. European Union ◦ ✅ Recognized as a novel food/dietary supplement. ◦ No formal inclusion in the European Pharmacopoeia, but officially permitted under EC/EFSA frameworks. USA ◦ ✅ Included in American Herbal Pharmacopoeia (AHP). ◦ ⚠️ Not yet in the USP–NF official drug compendium. Regulated as a dietary supplement.	Chinese & Korean Pharmacopoeias ◦ Historically focused on *C. sinensis*, but *C. militaris* is increasingly recognized in herbal/pharma spheres, especially cultivated strains. Thai & Vietnamese Pharmacopoeias ◦ ✅ Often include *C. militaris*, especially for research and supplement use; however, formal monograph details are sparse. European Union ◦ ⚠️ Not yet listed as a novel food or in Pharm Eur. Products containing *C. militaris* typically require novel-food approval or fall under THMPD (traditional herbal medicinal products) regulations. USA ◦ ✅ Included in AHP. ◦ ⚠️ Not yet in the USP–NF official compendia, but prevalent in supplement market.

**Table A17:** Health Claims (Supplements & Traditional Use) *.

Claim Category	*O. sinensis*	*C. militaris*
Energy and stamina	Traditional use for fatigue, high-altitude sickness	Claimed for fatigue and endurance, supported by more experimental data
Lung health/Respiratory health	Strong traditional use (asthma, COPD, bronchitis)	Similar claims; modern support based on anti-inflammatory effects
Kidney health/Tonic	Major use in Traditional Chinese Medicine (TCM)	Less emphasized traditionally, but included in general tonic claims
Libido/Sexual function	Used as an aphrodisiac and reproductive tonic	Also claimed, with more cordycepin-driven studies
Improving respiratory health	Broad claims in both systems	Often marketed more actively for immune health due to extract standardization
Anti-aging/Longevity	Central to its traditional appeal	Also marketed for longevity, supported by antioxidant research

*—Common Health Claims. Both species are marketed for: Acting as adaptogens to reduce fatigue and stress, enhancing energy and stamina, supporting immune function, and improving respiratory health.

**Table A18:** Use as Traditional Medicine and as Modern Dietary Supplements.

Category	*O. sinensis*	*C. militaris*
Traditional Chinese Medicine (TCM)	Used for chronic kidney disease, lung weakness, impotence, wasting diseases	Used in similar contexts, but less prestigious historically
Modern Supplements/Functional Foods	Rare, expensive; used in elite or luxury wellness products	Widely available; found in capsules, powders, drinks, etc.
Availability	Wild harvesting leads to scarcity and high prices; may contain contaminants or adulterants	Cultivated industrially; standardized extracts available; more sustainable
Research Base	Historically revered, but limited due to cost/availability	Extensive modern pharmacological studies due to ease of cultivation
Regulatory approval	Limited due to sourcing and consistency	More amenable to standardization and functional food use worldwide

**Table A19:** Indications for Use as medicines.

Indication	*O. sinensis*	*C. militaris*
Athletic performance	Traditional use	Supported by modern studies
Anti-aging & vitality	Strong cultural use	
Respiratory conditions	Common in TCM	Supported by modern studies
Cancer adjunct therapy	Limited evidence	Supported by modern studies
Immune modulation	Traditional use	Supported by modern studies
Chronic kidney disease	Traditional use	Supported by modern studies
lung weakness	Traditional use	
impotence	Traditional use	
Other chronic wasting diseases	Traditional use	

**Table A20:** Essential features.

Feature	*O. sinensis*	*C. militaris*
Cordycepin content	Minimal	High
Tradition (TCM)	Considered a superior tonic	Less traditional prestige
Cost	Extremely high	Affordable
Sustainability	Not sustainable	Fully cultivable
Main health focus	Lung, kidney, and general tonic	Immunity, energy, anti-cancer

**Table A21:** Definitions and Description of Essential Terms.

Term	Definitions and Description
Stress.	Stress is a systemic neuroendocrine response to perceived or actual challenges that threaten homeostasis. Stress is triggered by physical, psychological, metabolic, or social stressors, and mediated primarily by: (i)—Hypothalamic–pituitary–adrenal (HPA) axis (cortisol) and (ii)—Sympathetic nervous system (adrenaline, noradrenaline). The physiological role of stress is to mobilize energy, enhance alertness, and prioritize survival functions. Resolution: Stress hormones return to baseline after the threat passes and require intact feedback mechanisms.
Inflammation.	Inflammation is a localized, innate immune response to tissue injury, infection, or danger signals. Inflammation is triggered by pathogens, tissue damage, or toxins, mediated by immune cells, cytokines, and prostaglandins, and produces classic signs: redness, heat, swelling, pain, and loss of function. The physiological role of inflammation is to eliminate the cause of injury, remove damaged tissue, and initiate tissue repair. Resolution: inflammation is actively terminated by pro-resolving mediators (lipoxins, resolvins), returning tissue to baseline or near-baseline function.
The adaptive stress response.	The adaptive stress response is the organism’s capacity to respond to stressors efficiently and recover without damage. Adaptive stress response is characterised by: (i)—flexible activation and deactivation of stress pathways, (ii)—Involves cellular stress responses (heat shock proteins, antioxidant enzymes, autophagy), and (iii)—Maintains allostasis (stability through change). Physiological role: (i)—improves resilience, (ii)—enhances tolerance to future stressors, (iii)—prevents progression to pathology.
Pleiotropic activity.	In medicine, pleiotropic activity refers to a drug’s ability to produce multiple effects beyond its primary therapeutic purpose. This phenomenon, known as pleiotropy, can manifest as both additional therapeutic benefits and unwanted side effects. Understanding pleiotropic activity is crucial for drug repurposing, in which an existing drug is used to treat a new, distinct condition. It also influences the development of targeted therapies, as a treatment designed to address the root genetic cause of a condition could potentially alleviate several symptoms simultaneously. This approach offers a holistic method for managing complex genetic disorders, addressing the underlying biological defect rather than just symptoms.

Figure A1, Figure A2, Figure A3, Figure A4 and Figure A5 below show the chemical structures of primary and secondary metabolites of *O. sinensis* and *C. militaris*.
